# H3K27me3 Dynamic Turnover as a Gate Keeper of Defence Gene Expression in *Arabidopsis*

**DOI:** 10.3390/genes17080975

**Published:** 2026-08-19

**Authors:** Evangelia-Niki Pentari, Rory Osborne, Alonso Javier Pardal, Vardis Ntoukakis

**Affiliations:** 1 School of Life Sciences, University of Warwick, Coventry CV4 7AL, UK; evangelia.pentari@warwick.ac.uk; 2 School of Biosciences, University of Birmingham, Birmingham B15 2TT, UK; r.osborne@bham.ac.uk; 3 Department of Biochemistry, University of Cambridge, Cambridge CB2 1QX, UK

**Keywords:** pattern-triggered immunity, chromatin states, H3K27me3, histone demethylases

## Abstract

Background: Histone 3 lysine 27 tri-methylation (H3K27me3) is a chromatin mark typically associated with transcriptional repression. Histone demethylation, and particularly the removal of H3K27me3, has been linked to abiotic stress tolerance in plants. However, less is known about its role in biotic stress responses. Methods: We exploited immunity-related transcriptomics data combined with chromatin-state data to identify an association between chromatin modifications and plant immunity in *Arabidopsis thaliana*. We also measured the expression and H3K27me3 levels at immune-responsive loci, at *Col-0* and at histone deacetylase mutants. Results: We identified H3K27me3 as a mark correlated with the silencing of defence gene loci. Moreover, we showed that the expression of a subset of flg22-induced genes is repressed by H3K27me3 prior to elicitation, and that expression negatively correlates with the mark upon activation of immunity. Notably, our studies also revealed a role for the H3K27 demethylase REF6 in plant defence. Loss of REF6 allows ectopic H3K27me3 deposition at target genes, revealing that these loci are actively regulated by the demethylase. Conclusions: Our data provide insight into the regulation of plant immune responses through chromatin dynamics.

## 1. Introduction

Plants rely on a sophisticated and highly regulated immune system to restrict pathogen spread. Pattern-recognition receptors (PRRs) that recognise pathogen-associated molecular patterns (PAMPs) constitute the first line of molecular defence against invading pathogens. The recognition of PAMPs, such as the bacterial flagellum-derived peptide flg22, initiates a plethora of cellular responses, including production of reactive oxygen species, activation of mitogen-activated protein kinases (MAPKs), chromatin remodelling and activation of defence genes, collectively known as pattern-triggered immunity (PTI) [1,2,3]. This rapid transcriptional reprogramming is a hallmark of PTI. Approximately 2000 genes are differentially expressed in *Arabidopsis thaliana* within 30 min of elicitation by flg22 [4,5], a number that doubles within the first hour [5,6]. These responses are mediated by receptor-like cytoplasmic kinases, MAPK modules, calcium influx and calcium-dependent protein kinases (CDPKs), which collectively relay immune signals to the nucleus [7,8].

Interestingly, a substantial proportion of flg22-responding genes is shared with those induced by other PTI elicitors and endogenous plant danger signals, indicating convergence of signalling pathways on a common transcriptional programme [5,9,10]. However, signalling cascades alone cannot fully explain why some loci respond rapidly and robustly whereas others remain weakly expressed or non-responsive. In addition, defence-associated gene expression must be tightly constrained to prevent inappropriate immune activation and its associated fitness costs. Chromatin therefore provides an additional regulatory layer, integrating upstream immune signals with locus-specific control of transcriptional responsiveness.

Many chromatin modifications are closely associated with the transcriptional status [11,12,13,14]. Histone acetylation and H3K4 methylation are generally linked to active transcription, whereas H2B mono-ubiquitination and H3K36 tri-methylation correlate with productive elongation by RNA polymerase [15,16]. In addition to activating histone modifications, repressing chromatin marks can be recruited to flag silenced loci. Transcriptional repression in plants is commonly associated with DNA methylation, H3K9me2 in heterochromatin, and with H3K27me3 in many euchromatic genes [16,17]. H3K27me3 has been extensively studied for its role in plant development [18,19,20,21,22]. It is deposited by the Polycomb Repressive Complex 2 (PRC2) and removed by Jumonji C-domain histone demethylases (JmJC) [18,19,20,23] to repress or activate target loci, respectively. Reversible chromatin mark deposition is thus crucial for the control of adaptive gene expression.

Several chromatin modifications have previously been implicated in the regulation of plant immunity. Jaskiewicz et al. [24] observed a correlation between the H3K4me3 enrichment and defence gene priming. H3K9ac and H3K14ac, deposited by the histone acetyltransferase GCN5, have been shown to regulate salicylic acid (SA) accumulation following activation of immunity [25]. SA biosynthesis and signalling are also modulated through the histone deacetylase HDA6, under both basal and immune responsive conditions [26,27]. These examples underline the need for an efficient control of both the expression level and the timing of immune responses. Such regulation is likely to require coordinated action of multiple chromatin modifications.

Distinct combinations of chromatin modifications and genomic topological features, often associated with diverse transcriptional fates, are collectively defined as chromatin states. The first classification of chromatin into states was described in *Drosophila melanogaster* [28]. Later, Roudier et al. [16] identified four chromatin states in *Arabidopsis*, capturing the variation between actively transcribed, H3K27me3-repressed genes, intergenic and heterochromatic regions of high compactness. Since, more complex chromatin models accounting for multiple layers of epigenomic variation have been proposed. Sequiera-Mendes et al. [29] compiled a 9-state model that incorporates genome profiling of 11 chromatin marks, three histone variants, nucleosome density, GC content and DNA methylation data with a statistical model. More recently, an even more detailed, 36-chromatin state model has been reported, offering higher resolution on the cumulative effect of epigenetic marks in transcriptional control [30].

Among the different chromatin states, the poised chromatin state based on bivalent chromatin configurations has received particular attention [31,32,33]. This permissive configuration contains both activating and repressing marks, to maintain certain loci at a silenced yet readily active state. The term was first used to describe the co-occurrence of H3K27me3 and H3K4me3 histone modifications in mammalian stem cells, contributing to their pluripotency [31]. It has since been implicated in spermatogenesis [34], animal development [35], and cancer [36,37]. Similar poised configurations have also been described in plants, where they have been linked to transcriptional transitions during cold stress responses [38], vernalization [39] and root meristem development [40].

Although a specific chromatin state has not yet been clearly associated with plant immunity, emerging evidence suggests that immune-related transcriptional regulation can depend on the collaborative action of opposing chromatin marks. In soybean, for example, a mechanism involving antagonistic chromatin modifications has been shown to regulate immune receptor genes [33]. In *Arabidopsis*, the coordinated deposition of H3K27me3 and H3K18ac has been implicated in preventing the misregulation of metabolite production during PTI [41]. Together, these findings highlight the importance of tightly coordinated chromatin regulation in maintaining immune genes in an appropriately responsive, yet controlled, transcriptional state.

DNA methylation is another epigenetic mechanism that has been implicated in the control of immunity-responsive genes [42,43,44,45]. The methylation levels are dynamically regulated by the interplay between DNA methyltransferases and DNA demethylases [46], whose effects on transposable elements and defence-related genes have been associated with altered resistance to bacterial [42,43,47] and fungal pathogens [42,48,49]. Active DNA demethylation has been shown to restrict the spread of bacteria to the leaf vascular system, and has been proposed to contribute to defence gene priming by increasing promoter accessibility and facilitating the recruitment of the transcriptional machinery [43].

Here we show that a subset of defence genes is associated with a chromatin state defined by regions enriched in H3K4me3 and H3K4me2, which are marks correlated with active transcription, and H3K27me3, which is linked to gene repression. We further demonstrate experimentally that the expression of these genes is repressed by H3K27me3, and that their activation during immunity coincides with loss of this mark. Our analyses also implicate the H3K27me3 demethylase REF6 in immune regulation. REF6 maintains an H3K27me3-free environment at critical loci, ensuring prompt and robust gene upregulation during pathogen perception. Therefore, our findings support a role for chromatin dynamics in the transcriptional control of defence loci.

## 2. Materials and Methods

### 2.1. Transcriptomics Analysis

ATH1 GeneChip (Affymetrix, Santa Clara, CA, USA) Microarray data from Denoux et al. [10], generated after treatment of 10-day-old *Arabidopsis* seedlings with 1 μM flg22 or 50 μg/mL oligogalacturonides (OG), were used for the analysis. Stable expression across conditions and time points was defined when every fold-change for the four conditions (1 h and 3 h treatment with or without OG or flg22) was between 2 and −2. Next, to select highly and lowly expressed genes, this set of stable genes was sorted according to the averaged intensity signal for the four conditions and subdivided into two groups, one group of highly expressed genes, with those genes with average intensity (log_2_ intensity) greater than 5, and another group of lowly expressed genes, containing genes with average intensity smaller than 0.05.

A set of 1611 flg22-upregulated genes was selected from the microarray data (logFC > 2 and adjusted *p*-value ≤ 0.01). Gene coordinates were obtained from the TAIR10 annotation and converted to BED format. Genes shorter than 200 bp, as well as mitochondrial and chloroplast genes, were excluded from the analysis.

An empirical cumulative distribution function (ECDF) [50] was built on the microarray intensities of the differentially expressed genes from Denoux et al. [10], and identically distributed sets of genes were obtained through random sampling. The ECDF and random sampling were performed using R.

### 2.2. ChIP-Seq Analyses

Normalised chromatin state data (H3K4me2, H3K4me3, H3K36me3, H2Bub, H3K27me3, H3K27me1, histone H3 and DNA methylation) were gathered from the GEO database under the super-series accession number GSE24710 [16].

The H3K27me3-marked gene dataset was generated by analysing publicly available ChIP-seq data [51]. BigWig files were processed into 150 bp bins and the signal was computed across the gene bodies and regions spanning 1 kb upstream. Two independent thresholds were applied and genes satisfying a threshold in both ChIP-seq replicates were retained and pooled; genes were selected if enrichment spanned at least 600 bp (4 adjacent bins) with signal above the 92nd percentile (top 8%), or at least 450 bp (3 adjacent bins) with signal above the 95th percentile (top 5% of genes). Pooling both gene sets resulted in 3158 H3K27me3-marked genes.

Data from Cui et al. [52], associating REF6-binding peaks identified by ChIP-seq and REF6-binding motif analysis, were filtered to retain peaks containing at least one motif. These peaks were then assigned to genes based on overlap within the gene bodies. Since REF6 does not only regulate genes that contain a REF6-binding motif [52,53], we further included genes from the analysis of independent REF6 ChIP-seq data [51]. The bigWig files were analysed using a stringent threshold (~95–99th percentile across both replicates, corresponding to the top 5–1% enriched genes), to account only for highly enriched targets. The genes from the two lists were pooled, resulting in 3072 putative REF6 target loci.

For Venn diagram generation the R package ggVenn (Version 0.1.19) (https://yanlinlin82.github.io/ggvenn/, accessed on 6 June 2026) was used. TAIR (http://www.arabidopsis.org/, site accessed throughout the project), Araport (https://www.araport.org/), SALK primer T-DNA design (http://signal.salk.edu/tdnaprimers.2.html, site accessed throughout the project), PlantDHS (http://plantdhs.org/), String (https://string-db.org/), ArrayExpress (https://www.ebi.ac.uk/arrayexpress/), and BAR (http://bar.utoronto.ca/) databases were also accessed.

For the metaplots, genome-wide profiles of histone modifications were generated from publicly available ChIP–seq datasets for H3K27me3 (accession number: GSM5494301), H3K4me3 (GSM1225090), and H3K4me2 (Accession Number: DRX066756). Metagene profiles were generated using deepTools (Version 3.5.6). BigWig files were processed to achieve consistency of the chromosome names and were trimmed where necessary to match the TAIR10 chromosome size file. Gene bodies were scaled to an equal length of 1 kb and were aligned relative to the transcription start site (TSS) and transcription end site (TES), including 2 kb upstream and 1 kb downstream regions. Signal was computed in 50 bp bins using the computeMatrix scale-regions function with missing data treated as zero.

Genome browser tracks were generated using pyGenomeTracks (Version 3.9). Gene models were obtained from the Araport11/TAIR10 GTF annotations. Primer positions and TSS and TES locations were displayed using custom BED tracks. For minus-strand genes, loci were visualized at 5′ to 3′ orientation using the --decreasingXAxis option.

### 2.3. Plant and Microbe Materials and Growth Conditions

*A. thaliana* ecotype Columbia (*Col-0*) seeds were sowed on ‘*Arabidopsis* mix’ with Intercept and stratified for 2 to 3 days at 4 °C in darkness. Seeds were germinated and grown at a short photoperiod of 10 h light, 21 °C, 60% humidity. Two weeks after germination, seedlings were carefully transferred to individual pots. To obtain seeds, plants were transferred to a growth chamber with a 16 h light period.

For in vitro work, seeds were surface-sterilised by chlorine gas exposure: seeds in a seed storage (glassine) bag were kept for 4 h inside a sealed desiccator with a beaker containing 100 mL 10% sodium hypochlorite and 3 mL hydrochloric acid (37%). Seedlings were grown in sterile half (2.15 g/L) Murashige and Skoog medium (1/2 MS, Duchefa Biochemie, Haarlem, The Netherlands), with 1% sucrose, pH adjusted with KOH 1 M at 5.80 ± 0.02 and 0.5% Phytagel (Sigma-Aldrich, St. Louis, MO, USA) if solid medium required. Glufosinate at a final concentration of 20 µg/mL or nystatin 25 µg/mL, was added, if required, once the medium was autoclaved and cooled below 65 °C.

The SALK T-DNA insertion lines [54] for *Arabidopsis* were purchased from the Nottingham *Arabidopsis* Stock Centre (NASC). The *ref6-1* (SALK_001018) [55], and *elf6-3* (SALK_074694) mutants were genotyped using the genotyping primers indicated in Supplementary Table S1. The *fls*2 mutant described by Chinchilla et al. [56] was also used in this study.

### 2.4. Bacterial Infection Assay

*Pseudomonas syringae* strain DC3000 *∆avrPto ∆avrPtoB* was grown overnight in liquid culture to an optical density OD_600_ ~ 1.0. A 0.001 OD_600_ bacterial suspension (equivalent to 10^5^ colony-forming units/mL) was prepared in 10 mM MgCl_2_ for syringe-infiltration inoculations. Six 5-week-old plants per line were inoculated. At 3 days post-infection, 0.5 cm^2^ leaf discs were collected with a disc borer. Two leaf discs were collected per plant. Leaf discs were ground in 200 µL 10 mM MgCl_2_. To grind the plant tissue, two pulses of 28 Hz for 30 s were applied with a mixer mill (Tissue Lyser MM300, Retsch GmbH, Haan-Gruiten, Germany). Plant and bacterial suspension was then diluted up to 1 mL with 800 µL 10 mM MgCl_2_ and serial dilutions were plated on KB plates containing Rifampicin 100 µg/mL, Kanamycin 25 µg/mL, Spectinomycin 50 µg/mL. Each experiment, as described, was repeated 3 times on different days.

### 2.5. Flagellin Peptide Treatment

Seedlings grown in sterile conditions were treated with the small peptide flg22 from flagellin [57], sequence: QRLSTGSRINSAKDDAAGLQIA (EZBiolab Inc., Carmel, IN, USA). Seedlings were germinated and allowed to grow for 7 days in solid ½ MS, 1% sucrose, before transferring to liquid ½ MS, 1% sucrose. Two seedlings were transferred to each well containing 1 mL medium and were allowed to grow for another 7 days. After this adaptation period to the liquid medium, the remaining liquid was carefully removed from each well. Immediately, 1.5 mL of 100 nM flg22, ½ MS, 1% sucrose was added to the treatment wells, and fresh ½ MS, 1% sucrose, was added to the mock wells. Seedlings were harvested after 0.5, 1 or 2 h and flash frozen in liquid nitrogen for RNA or chromatin extraction.

### 2.6. RNA Extraction, cDNA Synthesis and qPCR

We followed protocols previously described in (Pardal, 2017) [58]. Briefly, plant tissue for RNA extraction was frozen in liquid nitrogen immediately after harvesting and kept at −80 °C until processing. Plant tissue was ground in liquid nitrogen, and 1 mL of TRIzol^®^ Reagent (Thermo Fisher Scientific, Waltham, MA, USA) was added to the powder and incubated at room temperature for 5 min; 200 µL of chloroform was added to the sample, mixed gently and incubated for another 5 min at room temperature. Samples were spun at 12,500× *g* for 20 min at 4 °C. The aqueous supernatant was carefully transferred to RNase-free tubes avoiding the aqueous interface. RNA was precipitated adding an equal volume of isopropanol and incubating for 3 h at −20 °C. Samples were spun at 16,800× *g* for 20 min at 4 °C. The supernatant was removed and the RNA pellet was rinsed twice with 1 mL 70% ethanol in DEPC water. Dried RNA pellets were re-suspended in 50 µL nuclease-free water by incubating at 65 °C for 5 min. RNA samples were treated with TURBO™ DNase (AM1907, Ambion, Thermo Fisher Scientific) following manufacturer’s instructions, and 5 µL of buffer and 1 µL of DNase enzyme were added to each RNA sample and incubated at 37 °C for 30 min. Then, 5 µL of the Inactivation Reagent was added, incubated at room temperature for 5 min and gently mixed by hand. After precipitating inactivating resin by centrifugation at 10,000× *g* for 1.5 min, 35 µL of clean RNA was transferred to a RNase-free tube. RNA quality was determined on a 1% agarose gel electrophoresis, and the concentration and purity were measured with a NanoDrop ND-1000 (Thermo Fisher Scientific).

For cDNA synthesis, 2 µg of RNA was reverse-transcribed with SuperScript™ II Reverse Transcriptase (18064, Thermo Fisher Scientific), following manufacturer’s specifications, using a primer for polyA tails (TTTTTTTTTTTTTTTTTTTVN). Final cDNAs were diluted with 40 µL nuclease-free water for a final volume of 60 µL. cDNAs were stored at −20 °C.

qPCR was performed with SYBR^®^ Green JumpStart™ Taq ReadyMix™ (S4438, Sigma-Aldrich), following manufacturer’s recommendations. Three technical repeats were used for each sample. A 384-well plate CFX384 Touch™ Real-Time PCR Detection System (Bio-Rad Laboratories, Watford, UK), and a 96-well plate Mx3005P qPCR System (Agilent Technologies, Santa Clara, CA, USA) were used. qPCR data were extracted for CT values (theoretical cycle to overcome a threshold) accepting automatically calculated thresholds and imported into an Excel file. Data were analysed using the ∆∆CT method [59]. The qPCR primers used are indicated in Supplementary Table S2. ACTIN 8 (ACT8) [60], B-TUBULIN (B-TUB) and TIP41-like family protein (TIP41) were used as controls. All qPCR primers were tested for efficiency on a standard curve with at least 6 template concentrations diluted 10-fold, accepting only efficiencies between 90 and 105%.

### 2.7. Chromatin Immuno-Precipitation (ChIP) and qPCR

Plant tissue treated with flagellin (as described above) was cross-linked and processed for chromatin immuno-precipitation (ChIP) following Engelhorn et al. [61] (personal communication). Plant tissue was cross-linked by adding 25 mL of cross-linking buffer (40 mM sucrose, 1 mM PMSF, 10 mM Tris/HCl pH 8.00, 1 mM EDTA and 1% formaldehyde) and vacuum-infiltration on ice for three rounds of 10 min. Excess formaldehyde was quenched by the addition of 1 mL 2 M glycine solution and vacuum-infiltration for an additional 5 min. Cross-linked tissue was rinsed twice in sterile water, dried with tissue paper and then frozen in liquid nitrogen.

Cross-linked tissue was thoroughly ground to fine powder in liquid nitrogen using a chilled pestle and mortar. Chromatin extraction used approximately 1 g of powder. Nuclei extraction was performed in 25 mL of Honda buffer (0.44 M sucrose, 1.25% ficoll, 2.5% dextran T40, 20 mM Hepes KOH pH 7.40, 10 mM MgCl_2_, 0.5% Triton X-100, 5 mM DTT, 1 mM PMSF, and 0.2% Protease Inhibitor Mix P), (39103, SERVA Electrophoresis GmbH, Heidelberg, Germany) [62]. Homogenised tissue was filtered with two layers of Miracloth (Millipore, Burlington, MA, USA), and nuclei were concentrated by centrifuging at 3000× *g* for 15 min at 4 °C. Iterative washes with Honda buffer and nuclei precipitation (1500× *g*, 4 °C, 7 min of centrifugation) ensured chloroplast depletion.

Clean nuclei (white pellet) were lysed and sonicated in 300 µL of Nuclei Lysis buffer (50 mM Tris/HCl pH 8.00, 10 mM EDTA, 1% SDS, 1 mM PMSF and 0.2% Plant Protease Inhibitors, Serva) per 1.5 mL tube. Sonication was conducted in ice-cold water, with Bioruptor^®^ (Diagenode, Seraing, Belgium) applying 6 cycles of 30 s “on”, 1 min “off” at high intensity (H) twice, changing icy water in between. Chromatin (diluted 10 times in TE buffer for measuring concentration) was quantified with a NanoDrop ND-1000 (Thermo Fisher Scientific), flash frozen in liquid nitrogen and stored at −20 °C unless immunoprecipitation was performed immediately after sonication. An aliquot from the pre-sonicated fraction as well as post sonicated were kept separate for on-gel fragment-size testing.

Sonicated chromatin samples were diluted in Nuclei Lysis Buffer for a final amount of 25 µg of chromatin in 50 µL per IP. A 10% sample was separated as DNA input (e.g., for three-antibody IPs, 15 µL were taken out of 150 µL). Then, chromatin samples were diluted 10 times in 450 µL of IP dilution buffer (1.1% Triton X-100, Sigma, 1.2 mM EDTA, 16.7 mM Tris/HCl pH 8.00, 167 mM NaCl, 0.2% Plant Protease Inhibitors). The required concentration of antibody was added, and the samples were incubated for 10 h at 4 °C and 12 rpm on an Intelli-Mixer Rotator Mixer RM-2 (EL-RM2L) (ELMI North America, Newbury Park, CA, USA). The antibodies used for ChIP-qPCRs are listed in Supplementary Table S3.

After antibody incubation, Protein A Agarose/Salmon Sperm DNA beads (16-157, Millipore) were added to the samples and incubated at 4 °C, gentle rotation, 12 rpm, overnight. Beads were washed five times in washing buffer (150 mM NaCl, 20 mM Tris HCl pH 8.00, 2 mM EDTA, 1% Triton X-100, 0.1% SDS, 1 mM PMSF, 0.2% Plant protease inhibitors) and eluted three times with 100 µL glycine elution buffer (0.1 M glycine, 0.5 M NaCl, 0.05% Tween-20, pH 3.80 adjusted with 37% HCl). The glycine elution buffer was made fresh every time and filter-sterilised. pH was neutralised by adding 150 µL of 1 M Tris/HCl pH 9.00. Input samples were treated in parallel from this point onwards, adding 300 µL of glycine elution buffer and 150 µL of Tris-HCl pH 9.

Samples were treated with 1.5 µL RNase A (10 mg/mL, R5125, Sigma-Aldrich) for 15 min at 37 °C, followed by 3 µL Proteinase K treatment overnight at 37 °C. Samples were de-crosslinked by incubating at 65 °C for 6 h. DNA samples were divided into two tubes and purified with a MinElute PCR Purification Kit (Qiagen, Hilden, Germany), following manufacturer’s instructions. Samples were eluted in 30 µL elution buffer, and DNA from corresponding IP samples were pooled together for a final volume of ~60 µL per sample. DNA samples were stored at −20 °C.

ChIP DNA samples were quantified by qPCR. Relative abundance of IP DNA was compared to input. qPCR was performed with SYBR^®^ Green JumpStart™ Taq ReadyMix™ (S4438, Sigma-Aldrich), following manufacturer’s recommendations. Three technical repeats were used for each sample. qPCR data were extracted for CT values (automatically calculated thresholds). As controls, *ACTIN* 7 (*ACT*7), an actively transcribed gene depleted of H3K27me3, and *SEPALLATA*3 (*SEP*3), a transcriptionally repressed locus enriched in H3K27me3 in the vegetative state [63], were used. Primers for genomic *FRK1* locus amplification were designed using *ref6* mutant data from Geshkovski et al. [64]. All ChIP-qPCR primers were tested for efficiency on a standard curve with at least six template concentrations diluted 10-fold, accepting only efficiencies between 90 and 105%. The ChIP-qPCR primers used are listed in Supplementary Table S4.

## 3. Results

### 3.1. Defence-Related Loci Are Associated with a Distinct Chromatin-State Signature

To test whether flagellin-responsive gene expression is correlated with specific chromatin marks, we analysed gene expression and chromatin state datasets. We used a four-chromatin state model [16] based on eight chromatin-associated marks across the *Arabidopsis* genome. Specifically, the model was generated by profiling H3K4me2/me3, H3K27me1/me3, H3K36me3, histone H2B ubiquitination (H2Bub), DNA methylation (5mC) and histone H3 enrichment (H3), as a proxy for nucleosome occupancy. To corroborate these chromatin state data, we also evaluated four previously published flg22-responsive gene expression profiles [10,16,65,66,67]. Among the available datasets, the Affymetrix ATH1 GeneChip microarray data by Denoux et al. [10] used the same experimental conditions as the ones used to generate the chromatin data [16] (Supplementary Table S5), making the inferences drawn from these data easier to interpret.

**Figure 1:**
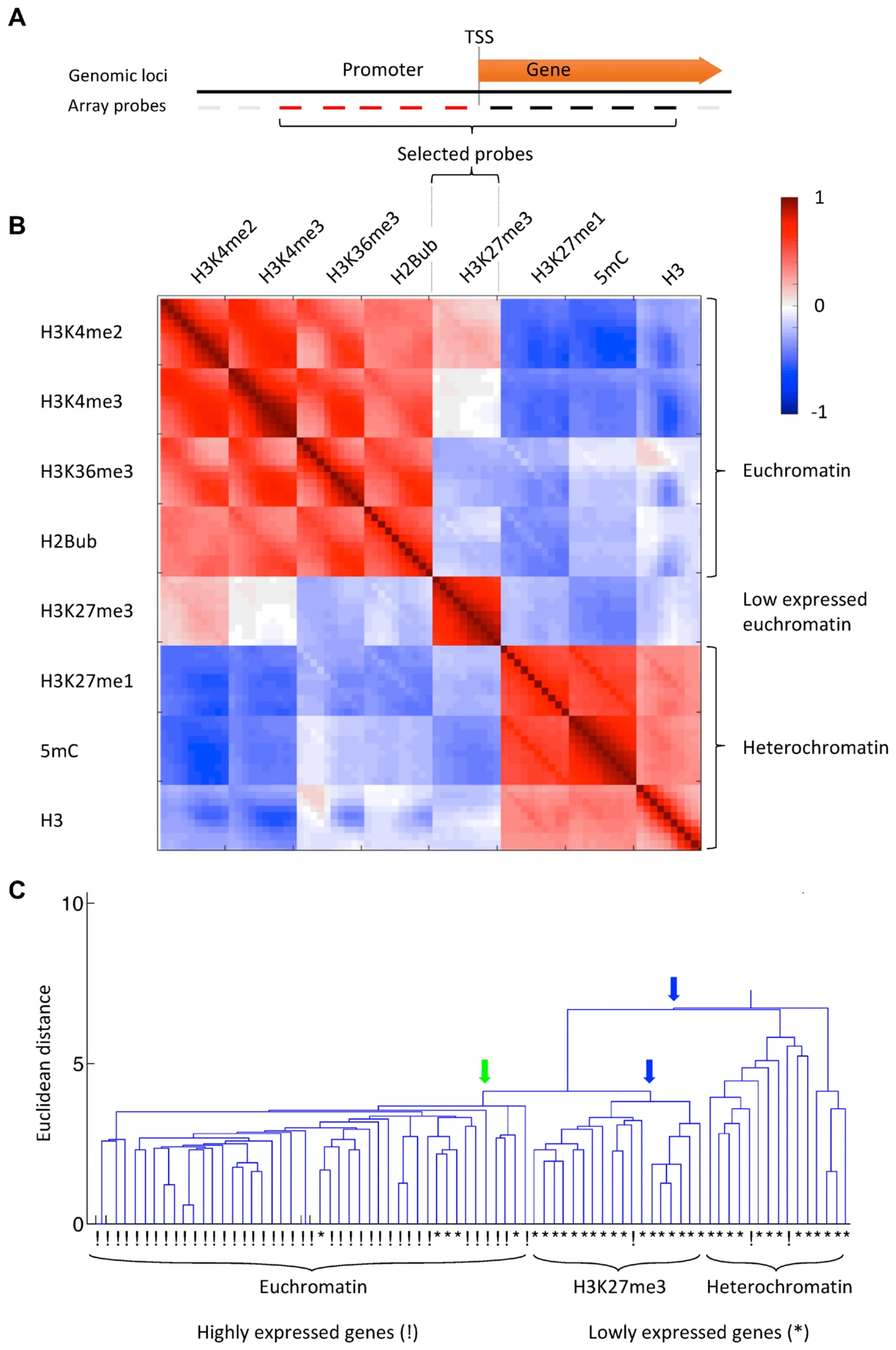
Chromatin marks correlate with each other and predict gene expression, singling out H3K27me3 as a euchromatic repressive mark. (**A**) Genomic positions of the tiling-array probes included in the analysis. For each gene, ten probes centred on the transcription start site (TSS) were selected: five upstream within the promoter region and five downstream within the transcribed region. (**B**) Pairwise correlation matrix of chromatin signals across 2497 stably expressed genes. The analysis included H3K4me2, H3K4me3, H3K36me3, H2B mono-ubiquitination (H2Bub), H3K27me3, H3K27me1, DNA methylation (5mC) and total histone H3 [10]. Each block represents correlations between signals measured at the ten TSS-centred probes shown in panel (**A**). Groups of marks associated predominantly with active euchromatin, H3K27me3-enriched lowly expressed euchromatin, or heterochromatin are indicated. (**C**) Hierarchical clustering of a representative subset of 80 stably expressed genes based on their chromatin profiles. Highly expressed genes are indicated by exclamation marks and lowly expressed genes by asterisks. Euclidean distance was used as the clustering metric. Arrows indicate the principal branch points separating active euchromatic genes and H3K27me3 from other lowly expressed genes enriched predominantly in heterochromatic features.

We next tested whether the correlation between the flg22-responsive gene expression and the chromatin states were directly comparable. For this purpose, stable expressing genes were defined from the expression dataset [10] by setting a threshold of less than two-fold gene expression change across all conditions (14,745 genes), resulting in 2497 stably expressed genes. Using the average intensity across replicates and treatments, stably expressed genes were then further divided into highly expressed stable genes (486 genes) and lowly expressed stable genes (2011 genes) (Supplementary Figure S1). We then correlated the chromatin marks of highly and lowly expressed stable genes [16], selecting five probes upstream and five probes downstream from the TSS (Figure 1A). In accordance with previously described results [16,29], the correlation matrix showed a high co-occurrence between histone modifications associated with expressed genes (H3K4me3, H3K4me2, H3K36me3 and H2Bub). This co-occurrence was also observed within the repressive marks (H3K27me1, DNA methylation, 5mC, and nucleosome abundance) (Figure 1A). Strikingly, H3K27me3 behaved independently from other repressive marks such as DNA methylation or H3K27me1, suggesting that Polycomb-mediated repression represents a distinct regulatory layer at defence gene loci (Figure 1A).

To investigate the relationship between chromatin modifications and gene expression patterns, genes were classified using Euclidean distances based solely on the histone modification profiles. Most genes were successfully grouped as highly or lowly expressed, suggesting a strong correlation between gene regulation and certain chromatin states (Figure 1B). While chromatin marks for actively transcribed genes were tightly clustered, marks for weakly expressed or inactive genes showed substantially higher variability. Lowly expressed genes formed two main clusters, one of which presented higher proximity to the active genes than to the other group of repressed genes (Figure 1B). This clustering suggests that active loci require a precise set of chromatin modifications for their regulation, but gene repression is associated with at least two independent chromatin marks, DNA methylation (5mC) and H3K27me3. Our meta-analysis verified that the chromatin state and expression datasets are directly comparable.

**Figure 2:**
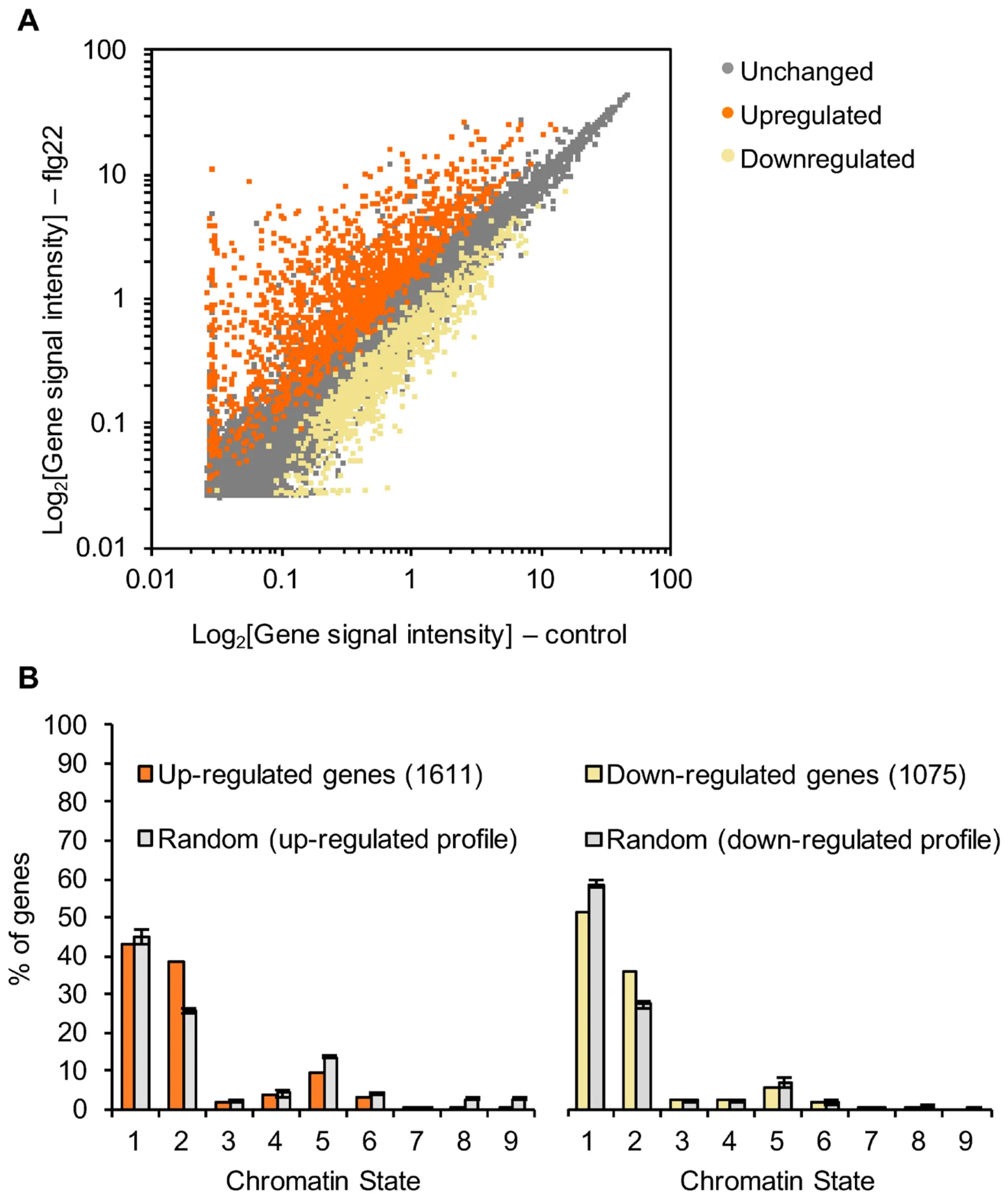
Distribution of flg22-responsive genes among *Arabidopsis* chromatin states. (**A**) Differentially expressed genes between control and 1 h after 1 μM flg22 [10]. (**B**) Distribution of 1611 upregulated and 1075 downregulated genes within the nine chromatin states defined by Sequeira-Mendes et al. [25]. Random control gene sets were matched to the pre-elicitation expression distributions of the responsive genes using an empirical cumulative distribution function. Ten independently sampled control sets were generated for each responsive-gene class. Bars show the percentage of genes assigned to each state; error bars for the random sets indicate standard deviations across the ten samples.

Next, we examined the chromatin-state distribution of flg22-responsive genes. Using a cutoff of fold-change >2 and *p*-value ≤ 0.01, we identified 1611 genes as significantly upregulated and 1075 as downregulated in response to flg22 (Figure 2A). We next mapped these genes onto the nine chromatin states defined by Sequeira-Mendes et al. [29] and compared their distribution across chromatin states with randomly generated gene sets as controls. These chromatin states represent combinations of histone modifications, histone variants, nucleosome occupancy and DNA methylation associated with different genomic environments, including active promoters, transcribed regions, Polycomb-associated chromatin and heterochromatin.

To ensure that differences between the flg22-responsive and random gene sets were not simply caused by differences in basal expression, the control sets were sampled to match as closely as possible the pre-elicitation expression distribution of the flg22-responsive genes. For this, we constructed an empirical cumulative distribution function (ECDF) [50] from the microarray intensity values of the gene set of interest and used it to generate random gene sets with a near-identical expression profile. This allowed us to compare chromatin-state distributions while controlling for basal gene expression.

Overall, the chromatin-state distribution of the flg22-responsive genes prior to immune activation was similar to that expected for random gene sets with comparable basal expression (Figure 2B). However, a higher-than-expected percentage of both upregulated and downregulated flg22-responsive genes was allocated to chromatin state 2. Chromatin state 2 is defined by regions enriched in the activating marks H3K4me3 and H3K4me2, which are correlated with active transcription, H3K27me3, which is associated with gene repression and H2A.Z [29], whose role in transcription is context dependent [68]. Interestingly, genes defined within this state display low levels of basal expression [29]. We also observed that the downregulated defence genes were underrepresented in chromatin state 1 compared to the randomly generated genes. Chromatin state 1 shares several features with state 2, including H3K4me2, H3K4me3 and H2A.Z, but lacks substantial H3K27me3 enrichment and instead contains higher levels of transcription-associated marks such as H3K36me3 and H2Bub [29]. These results indicate that the flg22-responsive genes present similarities to other expressed genes in chromatin mark deposition but are distinctive in key histone marks such as H3K27me3, a mark associated with low levels of transcription, or H3K4me3, which marks actively transcribed genes.

### 3.2. H3K27me3 and REF6 Define Distinct Subsets of flg22-Responsive Genes

Although H3K27me3 is typically associated with gene repression, several studies have suggested its presence in bivalent chromatin plays an important role in regulating gene expression, particularly in genes requiring rapid transcriptional activation [33]. Given that we observed a correlation between H3K27me3 and flg22-dependent gene expression, we compiled the 1611 flg22-upregulated genes (Figure 2), with a dataset of H3K27me3-marked genes under basal conditions [51] (Supplementary Table S6). This dataset was generated using two complementary enrichment thresholds to account for both the broad, moderate enrichment across genomic loci and the more localized regions exhibiting stronger enrichment. This approach identified 3158 H3K27me3-marked genes. The two datasets were derived from comparable experimental conditions, allowing for the direct comparison between PTI-responsive [10] and H3K27me3-marked gene populations [51].

The overlap between the two datasets revealed 118 genes that were flg22-responsive and marked by H3K27me3 in a naive state. This suggested that H3K27me3 is removed from these genes following elicitor perception, allowing for their expression. Given the known role of REF6 in H3K27me3 demethylation [18], we next included REF6 target genes to assess its potential involvement in defence gene derepression. To create a comprehensive set of candidate REF6 targets, genes associated with REF6-binding peaks that contained at least one REF6-binding motif within the peak region, as identified by Cui et al. [52], were combined with highly enriched genes from the analysis of an independent dataset [51], resulting in a list of 3072 REF6 target genes. Information on the datasets used to identify REF6 target genes is provided in Supplementary Table S6.

**Figure 3:**
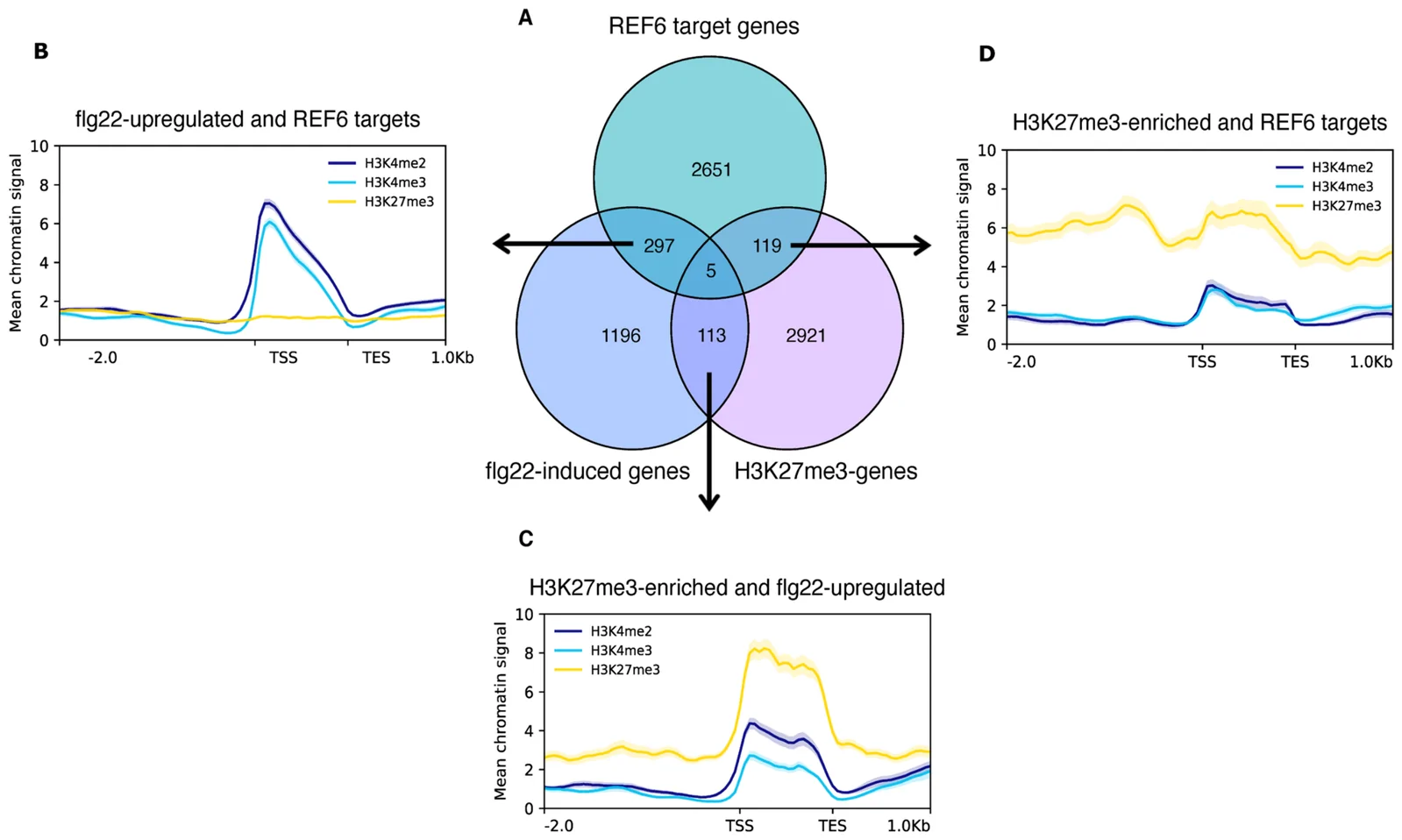
H3K27me3 and REF6 define subsets of flg22-induced genes with distinct chromatin composition. (**A**) Venn diagram illustrating the overlap between REF6 targets [51,52], flg22-upregulated [10] and H3K27me3-enriched genes [51] under basal conditions. (**B**–**D**) Metaplots of histone modifications (H3K27me3, H3K4me2 and H3K4me3) that define chromatin state 2 [29] across the gene subsets. (**B**) Intersection of flg22-upregulated genes and REF6 targets. The activating chromatin marks H3K4me2/me3 are enriched within the genomic loci, forming a peak at the TSS. (**C**) Intersection of flg22-upregulated and H3K27me3-enriched genes at naive condition, showing enrichment in H3K27me3 across their gene bodies. (**D**) Intersection of H3K27me3-enriched genes at naive conditions and REF6 targets, indicating a relatively homogeneous H3K27me3 deposition across both the gene body and intergenic regions.

The comparison of the three groups revealed three main gene clusters (Figure 3A). First, 297 flg22-induced genes were identified as REF6 targets not marked by H3K27me3 (Figure 3B), suggesting that REF6 keeps them at a poised-for-transcription state. Second, 113 genes were shared between the flg22-induced and H3K27me3-marked groups (Figure 3C). These genes were not identified as REF6 targets and may therefore require another demethylase to remove the repressing mark during activation of immunity. The third group consisted of 119 REF6 targets, methylated at naive conditions (Figure 3D). Only five genes were shared among all three datasets, indicating a limited overlap between REF6-targeted defence genes and H3K27me3-marked genes (Figure 3A).

Further analysis revealed that the H3K27me3 profiles differ between the methylated REF6-target genes that are not activated during PTI and the methylated flg22-responsive genes. H3K27me3-marked genes targeted by REF6 present a relatively uniform deposition of the H3K27me3 across their upstream regulatory regions, gene bodies and intergenic regions, with a noticeable drop before the transcription start site (TSS), where the activating chromatin mark deposition increases (Figure 3D). In contrast, flg22-responsive, H3K27me3-marked genes display a substantially higher H3K27me3 occupancy across both the TSS and the gene body, while the distribution of the mark is almost 4-fold lower at the promoter and 1 kb downstream from the transcription end site (TES) (Figure 3C). Our analysis also revealed that, at the loci of the flg22-responsive genes that possess reduced H3K27 methylation, H3K4me2/3 accumulate at higher levels (almost 3-fold) at the TSS and across the gene bodies (Figure 3B). This suggests an alternative regulation of those genes, indicative of a quick and robust transcriptional activation.

### 3.3. Flg22-Responsive Gene Expression Correlates with Local H3K27me3 Depletion

We next sought to experimentally validate our hypothesis that a group of flg22-upregulated genes is repressed by H3K27me3 under basal conditions. *WRKY75* and *CYP82C2* were selected as representatives of the 113 flg22-induced genes that we identified as H3K27me3-enriched from the bioinformatic analysis (Figure 3). Consistent with our analysis, H3K27me3 data for *Col-0* and the *ref*6 mutant [51] confirmed the REF6-independent deposition of H3K27me3 within the *CYP82C2* and *WRKY75* loci prior to flg22 elicitation (Supplementary Figure S2).

**Figure 4:**
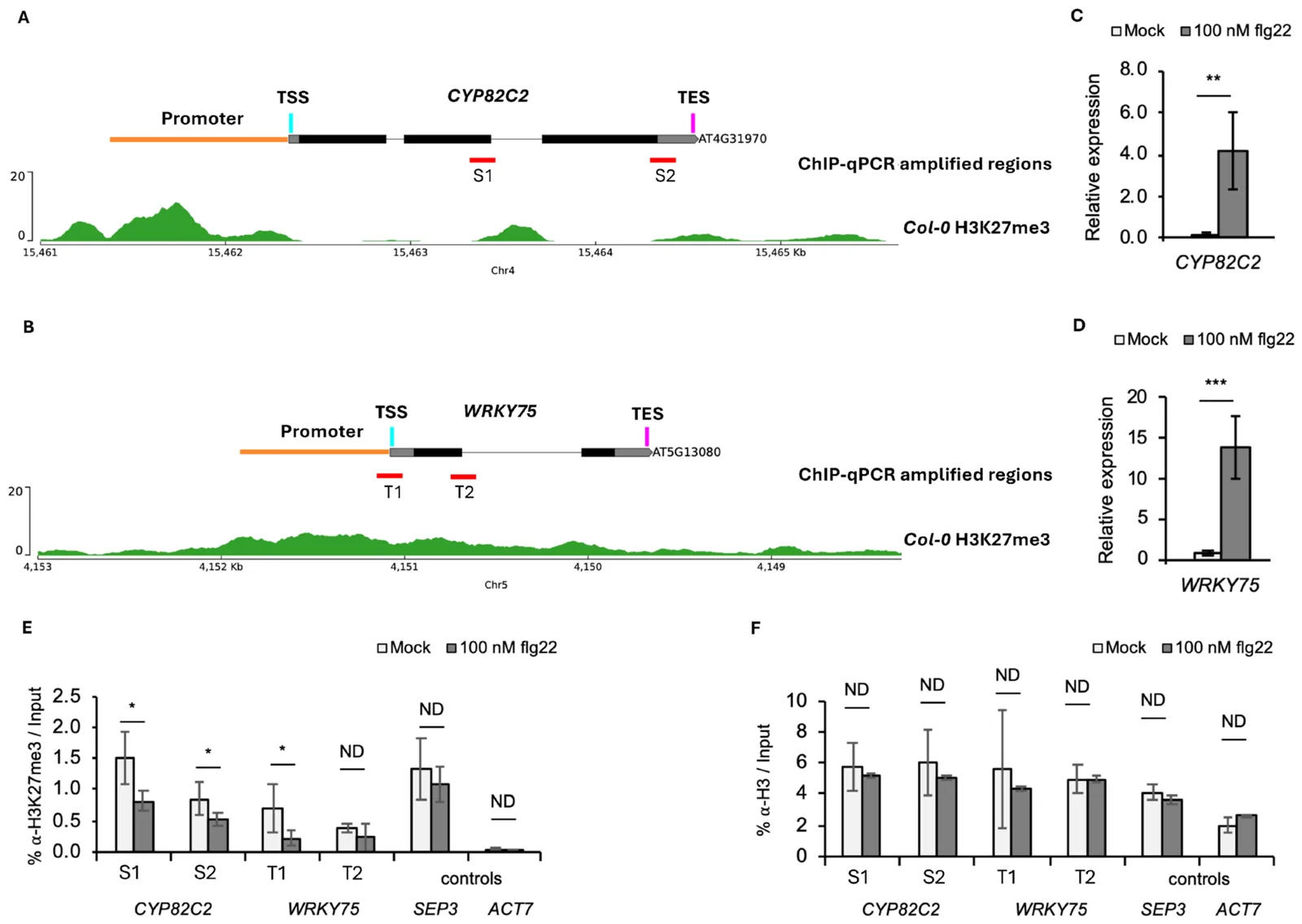
Dynamic anti-correlation between gene expression and H3K27me3 in response to flagellin at a subset of defence genes, not controlled by REF6. (**A**,**B**) Gene cartoons showing the deposition of H3K27me3 in *Col-0* (green tracks) at the *CYP82C2* (**A**) and WRKY75 (**B**) loci. ChIP-seq data from He et al. [51]. The transcription start sites (TSSs) and transcription end sites (TESs) are represented by the cyan and magenta vertical lines, respectively, and the promoter regions with orange bars. (**C**,**D**) qPCR analysis of *CYP*82*C*2 (**C**) and *WRKY*75 (**D**) gene expression in *Arabidopsis Col-0* seedlings 1 h after treatment with 100 nM flg22. (**E**,**F**) ChIP-qPCR analysis of the *CYP82C2* and *WRKY75* loci 1 h after treatment with 100 nM flg22. The ChIP-qPCR amplified regions S1 and S2 of *CYP82C2* and T1 and T2 of *WRKY*75 are indicated with red bars in (**A**) and (**B**), respectively. (**E**) ChIP anti-H3K27me3 for *CYP82C2* and *WRKY*75. (**F**) Control ChIP anti-H3 for *CYP82C2* and *WRKY*75. The *ACTIN* 7 (*ACT*7) and *SEPALLATA*3 (*SEP*3) genes were used as controls. Data shown are means of three biological replicates. Asterisks indicate: * *p* < 0.05, ** *p* < 0.01, *** *p* < 0.001; ND, no differences. Error bars represent standard deviations.

To understand the dynamics of H3K27me3 and its role in regulating the expression of *WRKY*75 and *CYP*82*C*2 during PTI, we treated *Arabidopsis* seedlings with 100 nM flg22 for 1 h and measured both gene expression and H3K27me3 enrichment. Both genes were significantly induced upon PTI activation (Figure 4C,D). This upregulation was mirrored with a decrease in H3K27me3 deposition at the same loci (Figure 4E,F).

Together, these data show 3HK27me3 removal during PAMP perception, suggesting the existence of a subset of defence genes that are enriched for H3K27me3 prior to elicitation and undergo H3K27me3 loss upon flg22 perception concomitant with their activation.

### 3.4. The REF6 Histone Demethylase Regulates the Expression of flg22-Responsive Genes

**Figure 5:**
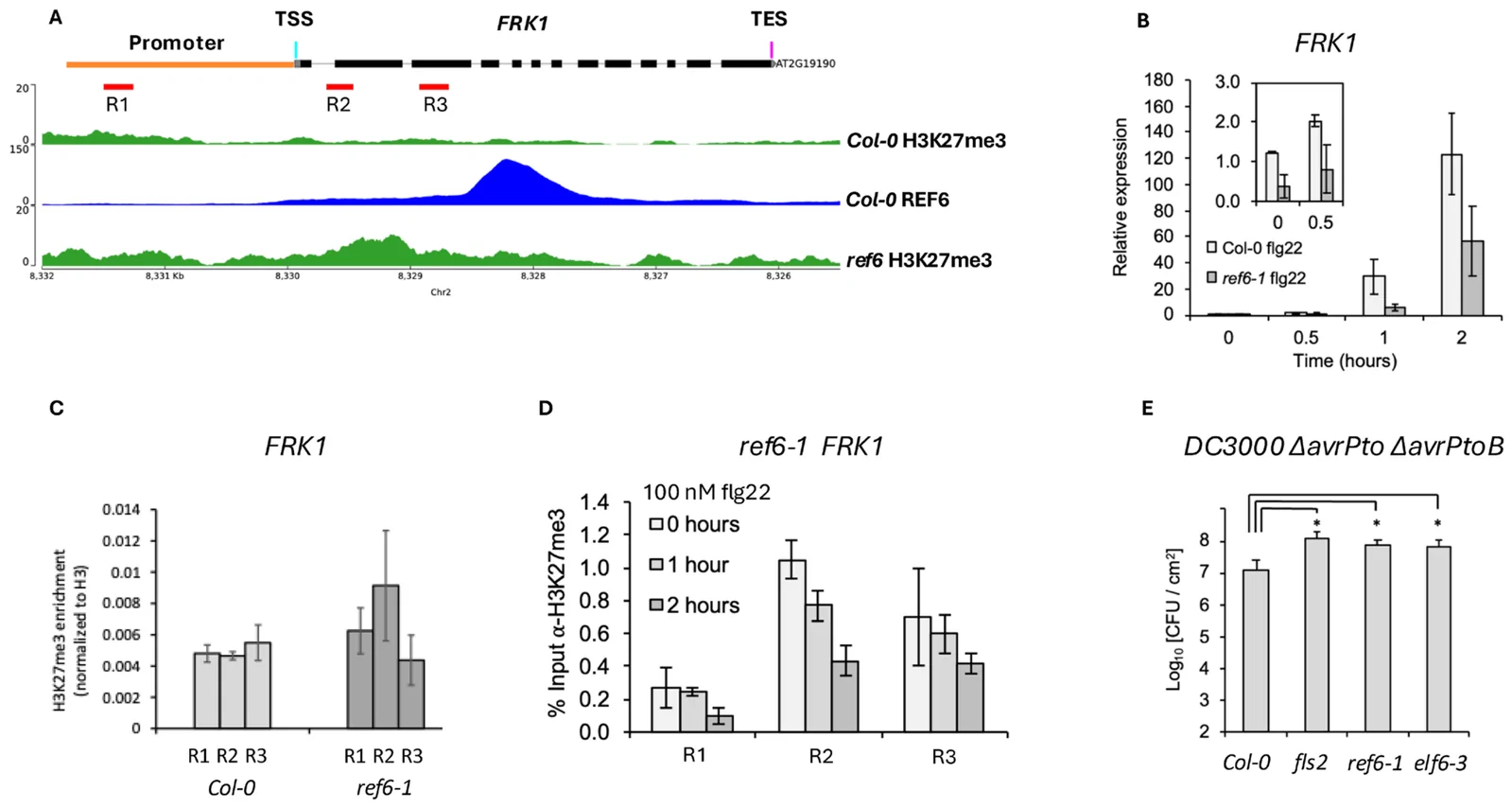
REF6 regulates *FRK1* expression under basal conditions by keeping its genomic locus depleted of H3K27me3. (**A**) Deposition of H3K27me3 and REF6 at the *FRK1* locus in *Col-0*, and of H3K27me3 in the *ref6-1* mutant. The *FRK1* locus contains low levels of the mark at normal conditions, which increase in the *ref*6 mutant. ChIP-seq data from He et al. [51]. The transcription start site (TSS) and transcription end site (TES) are represented by the cyan and magenta vertical lines, respectively, and the promoter region with the orange bar. (**B**) qPCRs of *ref6-1 FRK1* locus after 0.5, 1 and 2 h exposure to 100 nM flg22. flg22-induced gene upregulation of *FRK1* is impaired in the *ref6-1* mutant. *FRK1* expression is also diminished at the basal stage. *FRK1* transcript quantitation was assessed with two independent pairs of primers. Gene expression was quantified relative to reference genes *U-BOX*, *a-TUB* and *TIP*41. Error bars represent standard deviations. (**C**) ChIP-qPCR analysis of *FRK1* without treatment in *Col-0* and *ref6-1* mutant, and (**D**) after treatment with 100 nM flg22 in the mutant. (**E**) Histone demethylase mutants *ref6-1* and *elf6-3* are susceptible to *Pst* DC3000 *ΔAvrPto/AvrPtoB*. Five-week-old plants were sprayed with OD_600_ 0.001 and samples were harvested 3 dpi for bacterial re-isolation. *fls2* was used as a susceptible control. One representative experiment out of two biological repeats is shown. Statistical significance determined by a two-sided T-test assuming equal variances, *n* = 6, “*” indicates *p*-value < 0.05.

Our bioinformatic analysis also identified a subset of flg22-upregulated genes that are REF6 histone demethylase targets. REF6 could thus be responsible for their transcriptional priming in the absence of an elicitor. Among the 297 members of this group, we selected the immune-responsive gene *FRK1* to determine the role of REF6 in flg22-induced transcription. REF6 displayed a narrow binding peak within the *FRK1* locus, which is consistent with its low levels of methylation in *Col-0* (Figure 5A). Furthermore, the H3K27me3 deposition within the *FRK1* locus was notably increased in the *ref*6 mutant in comparison with the *Col-0* control plants (Figure 5A), supporting the hypothesis that REF6 actively removes H3K27me3 from the bodies of this subset of defence genes.

We next experimentally tested *FRK1* transcriptional activity using the REF6 demethylase mutant *ref6-1*. As expected, the flg22-induced upregulation of *FRK1* was notably impaired in the *ref6-1* mutant (Figure 5B). Consistent with this, the H3K27me3 levels were higher in the TSS of *FRK1* in the *ref6-1* mutant compared to *Col-0* (Figure 5C). Interestingly, flg22 elicitation led to a progressive decline of the mark (Figure 5D). These findings support a model in which REF6 establishes a poised state at flg22-responsive genes, which is putatively held in balance by the H3K27me3 writer PRC2.

### 3.5. Demethylase Loss Confers Susceptibility to Bacterial Infection

To directly test the role of REF6 in plant immunity, we assessed the resistance phenotype of the *ref6-1* mutant against the bacterial pathogen *P. syringae*. We selected a mutant strain lacking the effectors *AvrPto* and *AvrPtoB* (*Pst pathovar tomato* DC3000 *ΔavrPto ΔavrPtoB*), enabling the detection of more subtle immune phenotypes [69]. We also examined the closely related H3K27me3 demethylase mutant (*elf6-3*) [55], to determine whether the observed effects were specific to REF6 or reflected a broader role for H3K27me3 demethylases in immunity. In these experiments, we included the mutant of the FLS2 receptor [56] as a susceptible control. Both demethylase mutants were more susceptible than *Col-0* plants, supporting the role of REF6 as a positive regulator of immunity, and implicating the JmJC-demethylases as additional regulators of defence (Figure 5E).

**Figure 6:**
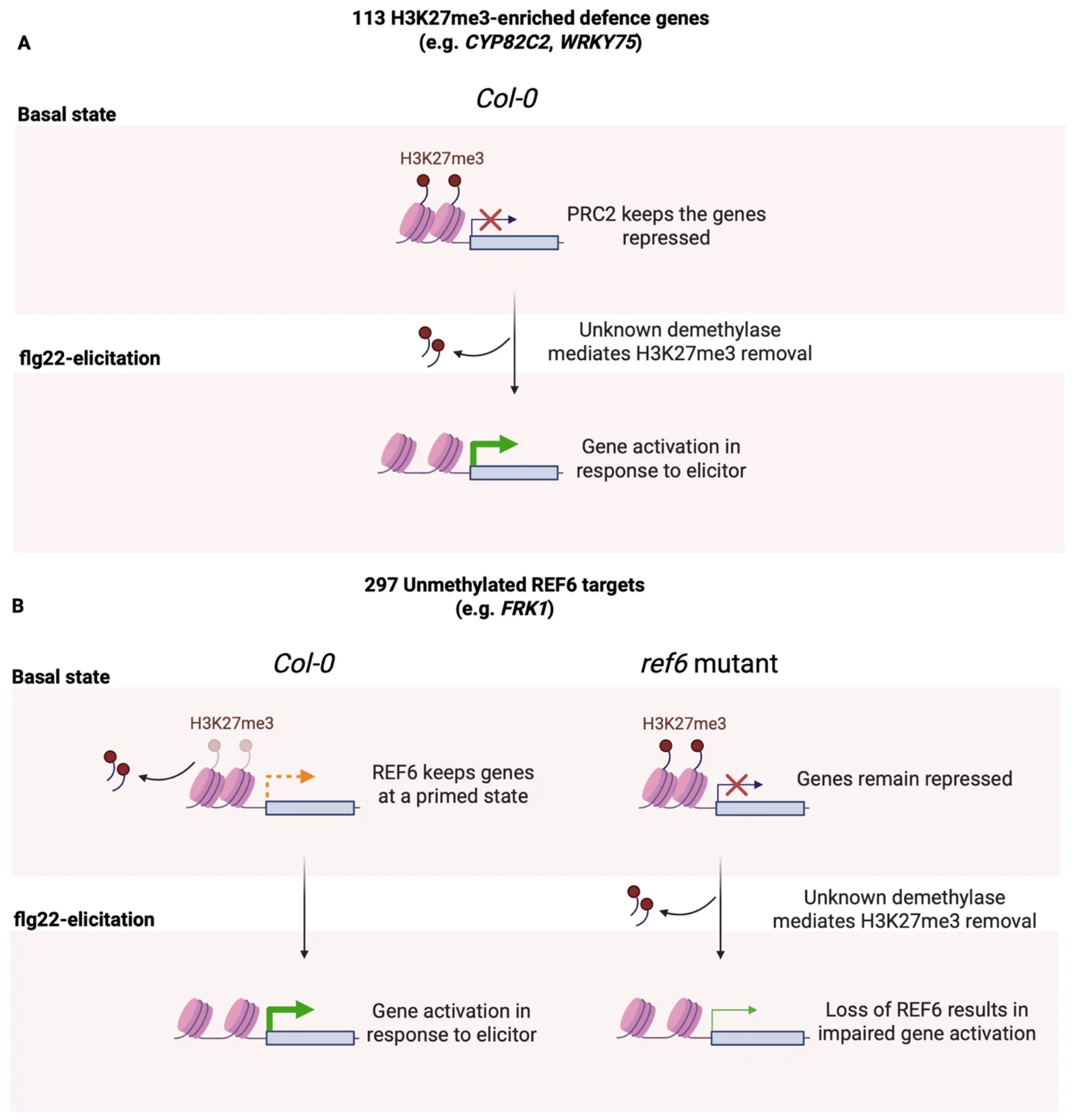
Proposed model illustrating the balance between PRC2 and REF6 at a subset of defence genes. (**A**) Regulation of 113 H3K27me3-enriched defence genes (e.g., *CYP82C2*, *WRKY*75). PRC2 adds H3K27me3, keeping the genes inactive under basal conditions. Upon flg22-perception, the mark is removed and the genes get activated. (**B**) REF6 antagonises PRC2 activity to keep target genes (e.g., *FRK1*) depleted of H3K27me3. The gene remains at a repressed yet poised state at pathogen-free conditions. Upon flg22-elicitation, *FRK1* becomes transcriptionally active. In the *ref*6 mutant background, *FRK1* accumulates H3K27me3 at the basal stage. Upon flg22-elicitation, *FRK1* is still induced, but at lower levels, indicating impaired activation in the absence of REF6, potentially due to higher H3K27me3. H3K27me3 levels also decrease on the *FRK1* locus through a REF6-independent mechanism.

In summary, our work shows that a subset of upregulated defence genes is marked and repressed by H3K27me3, a mark which is then removed during flg22-induced gene expression. Our data also imply that the ELF6 histone demethylase may be involved in this process. Furthermore, we identified a distinct subset of upregulated defence genes that are transcriptionally primed through the function of the REF6 histone demethylase, and therefore “prepared” for quick activation. Taken together, these findings put forward a dynamic mechanism in which multiple histone demethylases fine-tune transcriptional defence responses (Figure 6).

## 4. Discussion

Plant immune responses rely on finely tuned gene regulation to effectively deter and limit pathogen spread while minimising growth penalties. In this work, we investigated the relationship between chromatin state and flagellin-induced gene reprogramming in *Arabidopsis*. Our computational analyses underlined that, unlike heterochromatin-associated repressive marks, H3K27me3 is present within euchromatic regions and exhibits an important role in the expression of defence-related genes. Consistent with this observation, our experiments confirmed that flg22-induced transcription correlates with local H3K27me3 reduction. H3K27me3 is a repressive chromatin modification, known to mark genes that remain poised for future activation. Emerging evidence supports a role of H3K27me3 in modulating plant responses to biotic stress [27,70,71]. However, it is still unclear whether immune loci are regulated primarily by stable Polycomb repression that must be relieved upon elicitation, or whether some genes are subject to continuous cycles of H3K27 methylation and demethylation that maintain them in a low-expression but activation-competent state. It is also possible that the underlying mechanism is locus dependent.

In *Arabidopsis*, the H3K27 demethylases RELATIVE OF EARLY FLOWERING 6 (REF6/JMJ12) and EARLY FLOWERING 6 (ELF6/JMJ11) were first linked to developmental regulation and flowering-time control, including de-repression of the floral repressor *FLC* [18,55]. REF6 was later shown to bind to specific DNA motifs through its C-terminal zinc-finger domain, providing a mechanism for locus-specific targeting [52]. More recent studies have broadened this understanding by revealing that REF6 can establish or maintain H3K27me3-depleted chromatin states that confer transcriptional competence during developmental transitions such as seed germination [21,72,73], and can promote inducible gene expression in contexts such as thermomorphogenesis [51] and senescence [74]. Our data demonstrate that REF6 plays a similar role in regulating plant immunity. By actively maintaining a permissive chromatin state at selected flg22-responsive loci at pathogen-free conditions, REF6 enables their timely transcriptional activation upon elicitation. Loss of REF6 permits the ectopic PRC2-mediated H3K27me3 accumulation at these genes, leading to misregulation following immune activation.

Our results agree with the current understanding mode of H3K27me3 spreading, where PRC2, once it has nucleated a starting point, will continue spreading locally in a read-and-write fashion [75,76]. In this context, the *FRK1* locus is flanked by high levels of H3K27me3, which is consistent with the existing model of PRC2 processivity. In particular, PRC2 works by extending existing areas of repression (H3K27me3-marked) until it encounters a physical barrier, or, as we propose here, a counteracting enzyme—in this case REF6—which has similarly been proposed to function during development [21,77].

In accordance with the lack of substantial overlap between the REF6 targets, the flg22-upregulated, and the H3K27me3-enriched genes that we observed, Tomastikova et al. [27] also reported a limited number of REF6 targets among the genes that showed decreased H3K27me3 deposition following pathogen effector recognition. These findings further support the hypothesis that REF6 contributes to plant immunity mainly through establishing a permissive chromatin state prior to rather than during immune activation.

As REF6 and its paralogue ELF6 have previously been reported to exhibit functional redundancy [78,79], it is possible that the H3K27me3 depletion at defence target genes upon elicitation in the *ref6-1* mutant is mediated by ELF6. Indeed, our results demonstrate that the *elf6*-*3* mutant is also more susceptible to bacterial infection compared to the control plants, suggesting that ELF6 is involved in defence responses too. Additionally, ELF6 expression was shown to be induced following infection with *P. syringae* [80]. However, the exact mechanism by which ELF6 acts remains to be determined. In this regard, it remains to be tested which demethylases, if any, are actively recruited to loci such as *CYP82C2* or *WRKY75* during their transcriptional activation. Moreover, REF6 putative orthologues in rice have been shown to activate defence-associated genes, enhancing resistance to the bacterial pathogen *Xanthomonas oryzae* [81,82]. This finding indicates that the role of histone demethylases in plant immunity is conserved across flowering plants. Surprisingly, associated transcription factors that recruit the demethylases to their target genes have been reported to contribute to opposing transcriptional outcomes. Thus, an additional level of transcriptional regulation, through which the transcription factors counteract the effect of H3K27me3-mediated silencing, may be provided [21,77].

In addition to H3K27me3, other epigenetic marks, including DNA methylation and histone acetylation, are involved in the regulation of flg22-induced transcriptional responses. Indeed, DNA demethylation has been shown not only to facilitate, but also to be required for establishing efficient PTI responses [45]. Furthermore, the removal of H3K9ac by the histone deacetylase HD2B has been shown to lead to transcriptional silencing of certain PTI-responsive genes [83]. A similar effect was also observed by the histone deacetylase 19 (HDA19) upon infection with *P. syringae* DC3000 [84].

Collectively, our data support a model in which distinct subsets of pathogen-related genes are either repressed by H3K27me3 or maintained poised for transcription by the REF6 demethylase, enabling their activation at different times. Elicitor-responsive genes have been previously classified according to the timepoint at which they were first induced or repressed [5]. Additionally, bivalent chromatin modifications have previously been linked to timely defence gene expression [41]. It is therefore plausible that the difference between the activation of early and late PTI-responding genes is regulated by differences in chromatin composition prior to elicitation and dynamic changes in chromatin composition following the activation of immunity. Finally, the notion that distinct chromatin modifications may function at different stages of transcription has been proposed both in mammalian cells [85] and in Solanaceous plants in response to infection with the fungal pathogen *Botrytis cinerea* [86].

Taken together, these observations support the existence of a complex regulatory network for the timely and coordinated expression of *Arabidopsis* genes in response to pathogen cues. In addition to the post-translational modifications of key signalling components and the activation of downstream signalling cascades, our data show that reversible chromatin changes under basal conditions may affect the capacity of plants to mount defence gene responses upon pathogen perception. Additional layers of regulation are imposed at the transcriptional level, where the expression and recruitment of transcription factors and the efficiency of RNA polymerase II can impact the timing of gene activation [33,87] and likely act at the levels of ribosome assembly and protein synthesis [88]. Further investigation on how these regulatory layers define the transcription of defence genes will provide important insights into the balance between plant immunity and growth.

## Data Availability

The gene lists generated for the bioinformatics analyses are provided in Supplementary Datasets S1 and S2, as summarised in Supplementary Table S6. Supplementary Datasets S1 include gene lists derived from the publicly available transcriptomic dataset by Denoux et al., 2008 [10], publicly available H3K27me3 ChIP-seq data by He et al., 2022 [51], deposited in Gene Expression Omnibus (GEO) database under the accession numbers GSM5494301 and GSM5494302, the REF6-binding motif associated with peaks dataset by Cui et al., 2016 [52], and publicly available REF6 ChIP-seq data by by He et al., 2022 [51] (GSM5494309 and GSM5494310). The gene lists derived from the Venn diagram subgroups are provided in Supplementary Datasets S2. All other data supporting the findings of this study are available in the Supplementary Information.

## Supplementary Materials

The following supporting information can be downloaded at: https://www.mdpi.com/article/10.3390/genes17080975/s1, Figure S1: Classification of stably expressed genes-based average intensity; Figure S2: Gene cartoons showing the deposition of H3K27me3 in *Col-0* and *ref6* mutant backgrounds (green tracks) and ChIP-seq data for REF6 demethylase (blue track) in *Col-0*, at the *CYP82C2* (A) and *WRKY75* (B) loci; Table S1: List of genotyping primers; Table S2: List of qPCR primers; Table S3: List of ChIP-qPCR primers; Table S4: List of antibodies used for ChiP-qPCRs; Table S5: Transcriptomic and chromatin datasets considered for the bioinformatic analyses described in Figure 1 and Figure 2; Table S6: Description of datasets used for the bioinformatic analysis described in Figure 3.

## Abbreviations

The following abbreviations are used in this manuscript:

PRRs	Pattern-recognition receptors
PAMPs	Pathogen-associated molecular patterns
MAPKs	Mitogen-activated protein kinases
PTI	Pattern-triggered immunity
CDPKs	Calcium-dependent protein kinases
PRC2	Polycomb Repressive Complex 2
SA	Salicylic acid
ECDF	Empirical cumulative distribution function
TSS	Transcription start site
TES	Transcription end site
ChIP	Chromatin immunoprecipitation
qPCR	Quantitative polymerase chain reaction
MS	Murashige and Skoog medium
NASC	Nottingham Arabidopsis Stock Centre
REF6	Relative of Early Flowering 6
ELF6	Early Flowering 6
JmJC	Jumonji C-domain histone demethylases
OG	Oligogalacturonides

## References

[1] Boller T., Felix G. (2009). A Renaissance of Elicitors: Perception of Microbe-Associated Molecular Patterns and Danger Signals by Pattern-Recognition Receptors. Annu. Rev. Plant Biol..

[2] Macho A.P., Zipfel C. (2014). Plant PRRs and the Activation of Innate Immune Signaling. Mol. Cell.

[3] Ampntelnour L., Burton A., Ntoukakis V. (2026). How to Improve Crop Pathogen Resistance with Epigenetics. Phytopathol. Res..

[4] Thieffry A., López-Márquez D., Bornholdt J., Malekroudi M.G., Bressendorff S., Barghetti A., Sandelin A., Brodersen P. (2022). PAMP-Triggered Genetic Reprogramming Involves Widespread Alternative Transcription Initiation and an Immediate Transcription Factor Wave. Plant Cell.

[5] Bjornson M., Pimprikar P., Nürnberger T., Zipfel C. (2021). The Transcriptional Landscape of Arabidopsis Thaliana Pattern-Triggered Immunity. Nat. Plants.

[6] Winkelmüller T.M., Entila F., Anver S., Piasecka A., Song B., Dahms E., Sakakibara H., Gan X., Kułak K., Sawikowska A. (2021). Gene Expression Evolution in Pattern-Triggered Immunity within Arabidopsis Thaliana and across Brassicaceae Species. Plant Cell.

[7] Tena G., Boudsocq M., Sheen J. (2011). Protein Kinase Signaling Networks in Plant Innate Immunity. Curr. Opin. Plant Biol..

[8] Boudsocq M., Willmann M.R., Mccormack M., Lee H., Shan L., He P., Bush J., Cheng S.-H., Sheen J. (2010). Differential Innate Immune Signalling via Ca^2+^ Sensor Protein Kinases. Nature.

[9] Zipfel C., Kunze G., Chinchilla D., Caniard A., Jones J.D.G., Boller T., Felix G. (2006). Perception of the Bacterial PAMP EF-Tu by the Receptor EFR Restricts Agrobacterium-Mediated Transformation. Cell.

[10] Denoux C., Galletti R., Mammarella N., Gopalan S., Werck D., De Lorenzo G., Ferrari S., Ausubel F.M., Dewdney J. (2008). Activation of Defense Response Pathways by OGs and Flg22 Elicitors in Arabidopsis Seedlings. Mol. Plant.

[11] Jenuwein T., Allis C.D. (2001). Translating the Histone Code. Science.

[12] Wolffe A.P. (1994). Nucleosome Positioning and Modification: Chromatin Structures That Potentiate Transcription. Trends Biochem. Sci..

[13] Métivier R., Reid G., Gannon F. (2006). Transcription in Four Dimensions: Nuclear Receptor-Directed Initiation of Gene Expression. EMBO Rep..

[14] Felsenfeld G., Groudine M. (2003). Controlling the Double Helix. Nature.

[15] Zhang X., Bernatavichute Y.V., Cokus S., Pellegrini M., Jacobsen S.E. (2009). Genome-Wide Analysis of Mono-, Di- and Trimethylation of Histone H3 Lysine 4 in Arabidopsis Thaliana. Genome Biol..

[16] Roudier F., Ahmed I., Bérard C., Sarazin A., Mary-Huard T., Cortijo S., Bouyer D., Caillieux E., Duvernois-Berthet E., Al-Shikhley L. (2011). Integrative Epigenomic Mapping Defines Four Main Chromatin States in Arabidopsis. EMBO J..

[17] Zilberman D., Coleman-Derr D., Ballinger T., Henikoff S. (2008). Histone H2A.Z and DNA Methylation Are Mutually Antagonistic Chromatin Marks. Nature.

[18] Lu F., Cui X., Zhang S., Jenuwein T., Cao X. (2011). Arabidopsis REF6 Is a Histone H3 Lysine 27 Demethylase. Nat. Genet..

[19] Noh B., Lee S.-H., Kim H.-J., Yi G., Shin E.-A., Lee M., Jung K.-J., Doyle M.R., Amasino R.M., Noh Y.-S. (2004). Divergent Roles of a Pair of Homologous Jumonji/Zinc-Finger-Class Transcription Factor Proteins in the Regulation of Arabidopsis Flowering Time. Plant Cell.

[20] Gan E.-S., Xu Y., Wong J.-Y., Goh J.G., Sun B., Wee W.-Y., Huang J., Ito T. (2014). Jumonji Demethylases Moderate Precocious Flowering at Elevated Temperature via Regulation of FLC in Arabidopsis. Nat. Commun..

[21] Pan J., Zhang H., Zhan Z., Zhao T., Jiang D. (2023). A REF6-Dependent H3K27me3-Depleted State Facilitates Gene Activation during Germination in Arabidopsis. J. Genet. Genom..

[22] Yan Y., Zhu J., Qiu Q., Li J., Cao X., Deng X. (2025). The Arabidopsis Demethylase REF6 Physically Interacts with phyB to Promote Hypocotyl Elongation under Red Light. Proc. Natl. Acad. Sci. USA.

[23] Osborne R. (2026). Finding Balance: The Dynamic Interplay between H3K27me3 Writers and Erasers in Regulating Environmental Plasticity and Memory. New Phytol..

[24] Jaskiewicz M., Conrath U., Peterhänsel C. (2011). Chromatin Modification Acts as a Memory for Systemic Acquired Resistance in the Plant Stress Response. EMBO Rep..

[25] Kim S., Piquerez S.J.M., Ramirez-Prado J.S., Mastorakis E., Veluchamy A., Latrasse D., Manza-Mianza D., Brik-Chaouche R., Huang Y., Rodriguez-Granados N.Y. (2020). GCN5 Modulates Salicylic Acid Homeostasis by Regulating H3K14ac Levels at the 5′ and 3′ Ends of Its Target Genes. Nucleic Acids Res..

[26] Wang Y., Hu Q., Wu Z., Wang H., Han S., Jin Y., Zhou J., Zhang Z., Jiang J., Shen Y. (2017). HISTONE DEACETYLASE 6 Represses Pathogen Defence Responses in Arabidopsis Thaliana. Plant Cell Environ..

[27] Dvořák Tomaštíková E., Hafrén A., Trejo-Arellano M.S., Rasmussen S.R., Sato H., Santos-González J., Köhler C., Hennig L., Hofius D. (2021). Polycomb Repressive Complex 2 and KRYPTONITE Regulate Pathogen-Induced Programmed Cell Death in Arabidopsis. Plant Physiol..

[28] Filion G.J., van Bemmel J.G., Braunschweig U., Talhout W., Kind J., Ward L.D., Brugman W., de Castro I.J., Kerkhoven R.M., Bussemaker H.J. (2010). Systematic Protein Location Mapping Reveals Five Principal Chromatin Types in Drosophila Cells. Cell.

[29] Sequeira-Mendes J., Aragüez I., Peiró R., Mendez-Giraldez R., Zhang X., Jacobsen S.E., Bastolla U., Gutierrez C. (2014). The Functional Topography of the Arabidopsis Genome Is Organized in a Reduced Number of Linear Motifs of Chromatin States. Plant Cell.

[30] Liu Y., Tian T., Zhang K., You Q., Yan H., Zhao N., Yi X., Xu W., Su Z. (2018). PCSD: A Plant Chromatin State Database. Nucleic Acids Res..

[31] Bernstein B.E., Mikkelsen T.S., Xie X., Kamal M., Huebert D.J., Cuff J., Fry B., Meissner A., Wernig M., Plath K. (2006). A Bivalent Chromatin Structure Marks Key Developmental Genes in Embryonic Stem Cells. Cell.

[32] Lesch B.J., Page D.C. (2014). Poised Chromatin in the Mammalian Germ Line. Development.

[33] Jin L., Zhang Y., Guo J., Liu X., Lai Y., Huang X., Zou Y., Yan S., Dai X., Zhong Z. (2025). Epigenetically Poised Chromatin States Regulate PRR and NLR Genes in Soybean. aBIOTECH.

[34] Sin H.-S., Kartashov A.V., Hasegawa K., Barski A., Namekawa S.H. (2015). Poised Chromatin and Bivalent Domains Facilitate the Mitosis-to-Meiosis Transition in the Male Germline. BMC Biol..

[35] Macrae T.A., Fothergill-Robinson J., Ramalho-Santos M. (2023). Regulation, Functions and Transmission of Bivalent Chromatin during Mammalian Development. Nat. Rev. Mol. Cell Biol..

[36] Kumar D., Cinghu S., Oldfield A.J., Yang P., Jothi R. (2021). Decoding the Function of Bivalent Chromatin in Development and Cancer. Genome Res..

[37] Glancy E., Choy N., Eckersley-Maslin M.A. (2024). Bivalent Chromatin: A Developmental Balancing Act Tipped in Cancer. Biochem. Soc. Trans..

[38] Zeng Z., Zhang W., Marand A.P., Zhu B., Buell C.R., Jiang J. (2019). Cold Stress Induces Enhanced Chromatin Accessibility and Bivalent Histone Modifications H3K4me3 and H3K27me3 of Active Genes in Potato. Genome Biol..

[39] Gao Z., Li Y., Ou Y., Yin M., Chen T., Zeng X., Li R., He Y. (2023). A Pair of Readers of Bivalent Chromatin Mediate Formation of Polycomb-Based “Memory of Cold” in Plants. Mol. Cell.

[40] Li Q., Li J., Tang X., Chu C., Wang J., Zhou D.-X., Zhao Y. (2026). PLETHORA Transcription Factors Orchestrate Epigenetic Silencing of Bivalent Chromatin to Promote Root Meristem Development in Rice. Mol. Plant.

[41] Zhao K., Kong D., Jin B., Smolke C.D., Rhee S.Y. (2021). A Novel Bivalent Chromatin Associates with Rapid Induction of Camalexin Biosynthesis Genes in Response to a Pathogen Signal in Arabidopsis. eLife.

[42] López A., Ramírez V., García-Andrade J., Flors V., Vera P. (2011). The RNA Silencing Enzyme RNA Polymerase v Is Required for Plant Immunity. PLoS Genet..

[43] Yu A., Lepère G., Jay F., Wang J., Bapaume L., Wang Y., Abraham A.-L., Penterman J., Fischer R.L., Voinnet O. (2013). Dynamics and Biological Relevance of DNA Demethylation in Arabidopsis Antibacterial Defense. Proc. Natl. Acad. Sci. USA.

[44] López Sánchez A., Stassen J.H.M., Furci L., Smith L.M., Ton J. (2016). The Role of DNA (de)Methylation in Immune Responsiveness of Arabidopsis. Plant J..

[45] Huang M., Zhang Y., Wang Y., Xie J., Cheng J., Fu Y., Jiang D., Yu X., Li B. (2022). Active DNA Demethylation Regulates MAMP-Triggered Immune Priming in Arabidopsis. J. Genet. Genom..

[46] Law J.A., Jacobsen S.E. (2010). Establishing, Maintaining and Modifying DNA Methylation Patterns in Plants and Animals. Nat. Rev. Genet..

[47] Dowen R.H., Pelizzola M., Schmitz R.J., Lister R., Dowen J.M., Nery J.R., Dixon J.E., Ecker J.R. (2012). Widespread Dynamic DNA Methylation in Response to Biotic Stress. Proc. Natl. Acad. Sci. USA.

[48] Deleris A., Halter T., Navarro L. (2016). DNA Methylation and Demethylation in Plant Immunity. Annu. Rev. Phytopathol..

[49] Le T.-N., Schumann U., Smith N.A., Tiwari S., Au P.C.K., Zhu Q.-H., Taylor J.M., Kazan K., Llewellyn D.J., Zhang R. (2014). DNA Demethylases Target Promoter Transposable Elements to Positively Regulate Stress Responsive Genes in Arabidopsis. Genome Biol..

[50] Tucker H.G. (1959). A Generalization of the Glivenko-Cantelli Theorem. Ann. Math. Stat..

[51] He K., Mei H., Zhu J., Qiu Q., Cao X., Deng X. (2022). The Histone H3K27 Demethylase REF6/JMJ12 Promotes Thermomorphogenesis in Arabidopsis. Natl. Sci. Rev..

[52] Cui X., Lu F., Qiu Q., Zhou B., Gu L., Zhang S., Kang Y., Cui X., Ma X., Yao Q. (2016). REF6 Recognizes a Specific DNA Sequence to Demethylate H3K27me3 and Regulate Organ Boundary Formation in Arabidopsis. Nat. Genet..

[53] Tian Z., Li X., Li M., Wu W., Zhang M., Tang C., Li Z., Liu Y., Chen Z., Yang M. (2020). Crystal Structures of REF6 and Its Complex with DNA Reveal Diverse Recognition Mechanisms. Cell Discov..

[54] Alonso J.M., Stepanova A.N., Leisse T.J., Kim C.J., Chen H., Shinn P., Stevenson D.K., Zimmerman J., Barajas P., Cheuk R. (2003). Genome-Wide Insertional Mutagenesis of Arabidopsis Thaliana. Science.

[55] Crevillén P., Yang H., Cui X., Greeff C., Trick M., Qiu Q., Cao X., Dean C. (2014). Epigenetic Reprogramming That Prevents Transgenerational Inheritance of the Vernalized State. Nature.

[56] Chinchilla D., Bauer Z., Regenass M., Boller T., Georg F. (2006). TheArabidopsis Receptor Kinase FLS2 Binds Flg22 Determines Specificityof Flagellin Perception. Plant Cell.

[57] Bartels S., Lori M., Mbengue M., van Verk M., Klauser D., Hander T., Böni R., Robatzek S., Boller T. (2013). The Family of Peps and Their Precursors in Arabidopsis: Differential Expression and Localization but Similar Induction of Pattern-Triggered Immune Responses. J. Exp. Bot..

[58] Pardal A.J. (2017). Exploring the Role of Histone Marks and Chromatin Remodelling ATPases in Plant Immunity. PhD Thesis.

[59] Livak K.J., Schmittgen T.D. (2001). Analysis of Relative Gene Expression Data Using Real-Time Quantitative PCR and the 2(-Delta Delta C(T)) Method. Methods.

[60] Gimenez-Ibanez S., Boter M., Fernández-Barbero G., Chini A., Rathjen J.P., Solano R. (2014). The Bacterial Effector HopX1 Targets JAZ Transcriptional Repressors to Activate Jasmonate Signaling and Promote Infection in Arabidopsis. PLoS Biol..

[61] Engelhorn J., Turck F. (2010). Metaanalysis of ChIP-Chip Data. Methods Mol. Biol..

[62] Honda S.I., Hongladarom T., Laties G.G. (1966). A New Isolation Medium for Plant Organelles. J. Exp. Bot..

[63] Zhang T., Marand A.P., Jiang J. (2016). PlantDHS: A Database for DNase I Hypersensitive Sites in Plants. Nucleic Acids Res..

[64] Geshkovski V., Engelhorn J., Izquierdo J.-B., Laroussi H., Thouly C., Turchi L., Le Masson M., Thévenon E., Petitalot A., Simon L. (2026). The Dual trxG/PcG Protein ULTRAPETALA1 Modulates H3K27me3 and Directly Enhances POLYCOMB REPRESSIVE COMPLEX 2 Activity for Fine-Tuned Reproductive Transitions. Nat. Plants.

[65] Navarro L., Zipfel C., Rowland O., Keller I., Robatzek S., Boller T., Jones J.D.G. (2004). The Transcriptional Innate Immune Response to Flg22. Interplay and Overlap with Avr Gene-Dependent Defense Responses and Bacterial Pathogenesis. Plant Physiol..

[66] Zipfel C., Robatzek S., Navarro L., Oakeley E.J., Jones J.D.G., Felix G., Boller T. (2004). Bacterial Disease Resistance in Arabidopsis through Flagellin Perception. Nature.

[67] Frei dit Frey N., Garcia A.V., Bigeard J., Zaag R., Bueso E., Garmier M., Pateyron S., de Tauzia-Moreau M.-L., Brunaud V., Balzergue S. (2014). Functional Analysis of Arabidopsis Immune-Related MAPKs Uncovers a Role for MPK3 as Negative Regulator of Inducible Defences. Genome Biol..

[68] Gómez-Zambrano Á., Merini W., Calonje M. (2019). The Repressive Role of Arabidopsis H2A.Z in Transcriptional Regulation Depends on AtBMI1 Activity. Nat. Commun..

[69] Ntoukakis V., Mucyn T.S., Gimenez-Ibanez S., Chapman H.C., Gutierrez J.R., Balmuth L., Jones M.E., Rathjen J.P. (2009). Host Inhibition aBacterial Virulence Effector Triggers Immunity Infection. Science.

[70] Roy S., Gupta P., Rajabhoj M.P., Maruthachalam R., Nandi A.K. (2018). The Polycomb-Group Repressor MEDEA Attenuates Pathogen Defense. Plant Physiol..

[71] Osborne R., Labandera A.-M., Ryder A.J., Kanali A., Xu T., Akintewe O., Schwarze M.A., Morgan C.D., Hartman S., Kaiserli E. (2025). VRN2-PRC2 Facilitates Light-Triggered Repression of PIF Signaling to Coordinate Growth in Arabidopsis. Dev. Cell.

[72] Chen H., Tong J., Fu W., Liang Z., Ruan J., Yu Y., Song X., Yuan L., Xiao L., Liu J. (2020). The H3K27me3 Demethylase RELATIVE OF EARLY FLOWERING6 Suppresses Seed Dormancy by Inducing Abscisic Acid Catabolism. Plant Physiol..

[73] Sato H., Santos-Gonzalez J., Köhler C. (2021). Combinations of maternal-specific repressive epigenetic marks in the endosperm control seed dormancy. eLife.

[74] Wang X., Gao J., Gao S., Song Y., Yang Z., Kuai B. (2019). The H3K27me3 Demethylase REF6 Promotes Leaf Senescence through Directly Activating Major Senescence Regulatory and Functional Genes in Arabidopsis. PLoS Genet..

[75] Lee C.-H., Yu J.-R., Kumar S., Jin Y., LeRoy G., Bhanu N., Kaneko S., Garcia B.A., Hamilton A.D., Reinberg D. (2018). Allosteric Activation Dictates PRC2 Activity Independent of Its Recruitment to Chromatin. Mol. Cell.

[76] Sauer P.V., Pavlenko E., Cookis T., Zirden L.C., Renn J., Singhal A., Hunold P., Hoehne-Wiechmann M.N., van Ray O., Kaschani F. (2024). Activation of Automethylated PRC2 by Dimerization on Chromatin. Mol. Cell.

[77] Yan W., Chen D., Smaczniak C., Engelhorn J., Liu H., Yang W., Graf A., Carles C.C., Zhou D.-X., Kaufmann K. (2018). Dynamic and Spatial Restriction of Polycomb Activity by Plant Histone Demethylases. Nat. Plants.

[78] Yang H., Howard M., Dean C. (2016). Physical Coupling of Activation and Derepression Activities to Maintain an Active Transcriptional State at FLC. Proc. Natl. Acad. Sci. USA.

[79] Crevillén P. (2020). Histone Demethylases as Counterbalance to H3K27me3 Silencing in Plants. iScience.

[80] Lewis L., Polanski K., De Torres-Zabala M., Jayaraman S., Bowden L., Moore J., Penfold C., Jenkins D.J., Hill C., Baxter L. (2015). TranscriptionalDynamics Driving MAMP-Triggered Immunity Pathogen Effector-Mediated Immunosuppression Arabidopsis Leaves Following Infection withPseudomonas Syringae Pv Tomato DC3000. Plant Cell.

[81] Li T., Chen X., Zhong X., Zhao Y., Liu X., Zhou S., Cheng S., Zhou D.-X. (2013). Jumonji C Domain Protein JMJ705-Mediated Removal of Histone H3 Lysine 27 Trimethylation Is Involved in Defense-Related Gene Activation in Rice. Plant Cell.

[82] Hou Y., Wang L., Wang L., Liu L., Li L., Sun L., Rao Q., Zhang J., Huang S. (2015). JMJ704 Positively Regulates Rice Defense Response against Xanthomonas Oryzae Pv. Oryzae Infection via Reducing H3K4me2/3 Associated with Negative Disease Resistance Regulators. BMC Plant Biol..

[83] Latrasse D., Jégu T., Li H., de Zelicourt A., Raynaud C., Legras S., Gust A., Samajova O., Veluchamy A., Rayapuram N. (2017). MAPK-Triggered Chromatin Reprogramming by Histone Deacetylase in Plant Innate Immunity. Genome Biol..

[84] Kim K.-C., Lai Z., Fan B., Chen Z. (2008). Arabidopsis WRKY38 and WRKY62 Transcription Factors Interact with Histone Deacetylase 19 in Basal Defense. Plant Cell.

[85] Gates L.A., Shi J., Rohira A.D., Feng Q., Zhu B., Bedford M.T., Sagum C.A., Jung S.Y., Qin J., Tsai M.-J. (2017). Acetylation on Histone H3 Lysine 9 Mediates a Switch from Transcription Initiation to Elongation. J. Biol. Chem..

[86] Crespo-Salvador Ó., Sánchez-Giménez L., López-Galiano M.J., Fernández-Crespo E., Schalschi L., García-Robles I., Rausell C., Real M.D., González-Bosch C. (2020). The Histone Marks Signature in Exonic and Intronic Regions Is Relevant in Early Response of Tomato Genes to Botrytis Cinerea and in miRNA Regulation. Plants.

[87] Mayer A., Landry H.M., Churchman L.S. (2017). Pause & Go: From the Discovery of RNA Polymerase Pausing to Its Functional Implications. Curr. Opin. Cell Biol..

[88] Yu D.-T., Xie S.-S., Duan C.-G. (2026). Chromatin Accessibility and Histone Landscapes Define Immune Signal-Specific Transcriptional Reprogramming in Arabidopsis. aBIOTECH.

