# Supplementary material for: H3K27me3 Dynamic Turnover as a Gate Keeper of Defence Gene Expression in Arabidopsis
Source: Genes (Basel). 2026 Aug 19;17(8):975. doi: 10.3390/genes17080975 (PMC13512190; doi:10.3390/genes17080975)
Supplement: Supplementary file 1 [file genes-17-00975-s001.zip › genes-4439870-supplementary_AJP.pdf]

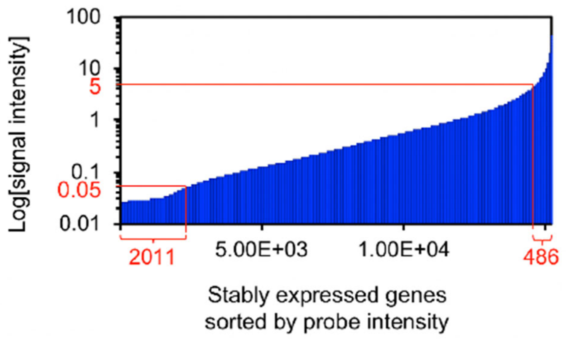

Supplementary Figure S1. Stably expressed genes (14,745 genes with less than 2-fold gene expression change between every replicate out of three, treatment; mock, flg22 and OG, and time point; 1 and 3 hours) [1]. Genes (on X axis) are sorted in ascendant order according to average signal intensity (Y axis). Selected lowly expressed genes (probes with less than 0.05 log intensity, bottom 2011), and highly expressed genes (probes with an intensity greater than 5 log, top 486 genes) for further analysis.

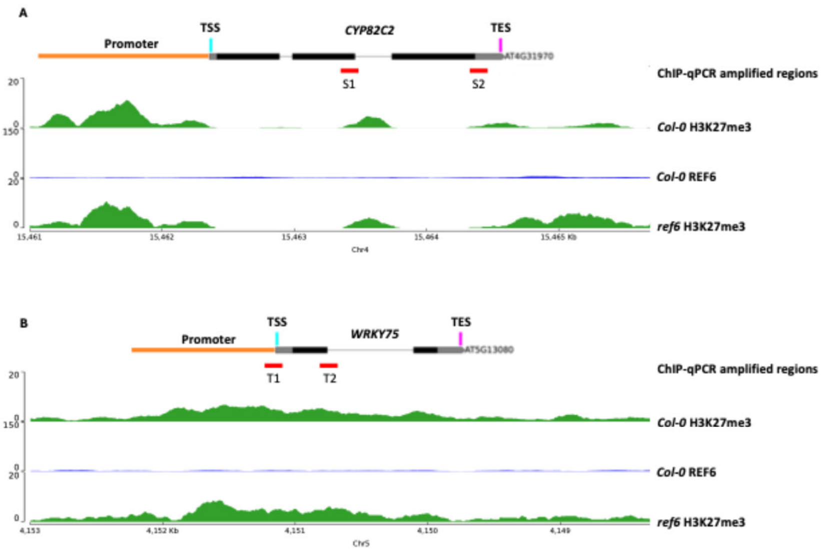

Supplementary Figure S2. Gene cartoons showing the deposition of H3K27me3 in Col-0 and *ref6* mutant backgrounds (green tracks) and ChIP-seq data for REF6 demethylase (blue track) in Col-0, at the *CYP82C2* (A) and *WRKY75* (B) loci. ChIP-seq data from He et al. [2].

Supplementary Table S1. List of genotyping primers.

Name	Gene	Sequence (5′ to 3′)	Description
LBb1.3	Left T-DNA insert border	ATTTTGCCGATTTCGGAAC	Genotyping SALK lines
RB1.2	Right T-DNA insert border	CTCCTTCAACGTTGCGGTTCTG	Genotyping SALK lines
N654971_LP	AT3G48430	TCATATACAAGGCGTTCGGTC	Line *ref6-1*, SALK_001018
N654971_RP	AT3G48430	CAGTTGCAACTCTGGAGAAGG	Line *ref6-1*, SALK_001018
N666435_LP	AT5G04240	ACGTCAATGCGGTAATCATTC	Line *elf6-3*, SALK_074694
N666435_RP	AT5G04240	TTTGCAGATCCCATTGCTTAC	Line *elf6-3*, SALK_074694

Supplementary Table S2. List of qPCR primers.

Primer name	sequence	Primer info
qCYP82C2_F	CTGTTTTCGGGTTTGCGCCT	AT4G31970
qCYP82C2_R	GCTTTCCGGCCACCATTCTC	
qCYP82C2_Fzg	AATCTACCTGCCTGGCACTG	AT4G31970, Zipfel lab [9,52]
qCYP82C2_Rzg	GAGAAATGGCCCATGTAAGG	
qWRKY75_F	GTGGATTTCTCGATGGGATGCG	AT5G13080
qWRKY75_R	TGCGTTTCAAACAAGGAGCCAA	
qFRK1-F	ATCTTCGCTTGGAGCTTCTC	AT2G19190, Zipfel lab [9]
qFRK1-R	TGCAGCGCAAGGACTAGAG	
ACT8-F	CCAGTGGTCGTACAACCGGTA	AT1G49240, Solano's group [47]
ACT8-R	TAGTTCTTTTCGATGGAGGAGCTG	AT1G49240,Solano's group [47]
qaTUB_F	TACACCAACCTCAACCGCCT	apha tubuline 4 AT1G04820
qaTUB_R	TGGGGCATAGGAGGAAAGCA	apha tubuline 4 AT1G04820
qTIP41_F	GTTGGTGCCTCATCTTCGCC	AT4G34270
qTIP41_R	GAACTGGCTGACAATGGAGTGT	AT4G34270

Supplementary Table S3. List of ChIP-qPCR primers.

Primer name	Sequence	Description (Amplified region)
FRK1_Prmtr_F	GCGAGGAAGAGGAAGTGAGG	R1
FRK1_Prmtr_R	GTTGTAGAAGCCGCGGTAGT	R1
FRK1_TSS_2_F	CACTTGATTGAAACTGAGCTGCA	R2
FRK1_TSS_2_R	CCGGATGATTCTAGCTACAACGA	R2
FRK1_5_b_F	TCGGGCGGTCTGAAACTAAG	R3
FRK1_5_b_R	TGACCGTATATGGACACCGC	R3
CYP82C2_3p_F	CACAGGAGAAGCAAAAGAGTTCG	S1
CYP82C2_3p_R	TGAAGGAAACGAGCAAGACCTAA	S1
CYP82C2_5p3_F	TGATGGTGAGAATGGTGGCC	S2
CYP82C2_5p3_R	AATTTGCGACGCCCTTTCTG	S2
WRKY75_5p_F	TCTAGGGCCGATGTGAATGA	T1
WRKY75_5p_R	GGCCGTCAAGAACAACAAGT	T1
WRKY75_TSS_1_F	GACAGACCGATCCACCTGAA	T2
WRKY75_TSS_1_R	CGCCTCTTCGTCTTTGACTT	T2
ACT7_F	CGTTTCGCTTTCCTTAGTGTTAGCT	*Actin 7*, AT5G09810, Primers for ChIP from Engelhorn [48]
ACT7_R	AGCGAACGGATCTAGAGACTCACCTT	*Actin 7*, AT5G09810, Primers for ChIP from Engelhorn [48]
SEP3_3p_F	Acattagcgtcaattcaaaaccca	*SEPALLATA3*, AT1G24260
SEP3_3p_R	gtttccaccttttgattctgggg	*SEPALLATA3*, AT1G24260
SEP3_5p_F	CTTGAGATACTCCTGCTGGCTAC	*SEPALLATA3*, AT1G24260
SEP3_5p_R	CACCAGAACCCAATGTGCCT	*SEPALLATA3*, AT1G24260

Supplementary Table S4. List of antibodies used for ChiP-qPCRs.

Target	Reference number	Volume used per IP
Rabbit polyclonal to Histone H3	ab1791 Abcam	1 µL
Rabbit polyclonal to H3K27me3	07-449 Millipore	9 µL

Supplementary Table S5. Transcriptomic and chromatin datasets considered for the bioinformatic analyses described in Figures 1 and 2. Datasets used in the analyses are underlined.

**Authors**	Navarro et al., 2004 [51]	Zipfel et al., 2004 [52]	**Denoux et al., 2008** [10]	Frei dit Frey et al., 2014 [53]	**Roudier et al., 2011** [12]
**flg22 treatments**	0.5 & 1 h	1 h	1 h	0.5 h	Untreated
**Tissue**	Protoplasts	leaf	Seedling	Seedling	Seedling
**Age**	7 days subculture	5-6 weeks	10 days	13 days	10 days
**Photoperiod**	NA	8 h light	16 h light	16 h light	16 h light
**Background**	Ler	Ler	Col-0	Col-0	Col-0
**Upregulated genes**	93	967	1,611	1,529	NA

Supplementary Table S6. Description of datasets used for the bioinformatic analysis described in Figure 3.

**Dataset**	**Type of original data**	**Threshold used**	**Number of genes**	**References**	**Accession number**
**flg22-upregulated**	Microarray data	logFC> 2 and adjusted p-value≤ 0.01	1,611	Denoux et al., 2008 [1]	
**H3K27me3 genes**	ChIP-seq data	≥ 450 bp with signal at the 95th percentile in both replicates (2631 genes)	3,158	He et al., 2021 [2]	GSM5494301<br>GSM5494302

		≥ 600bp with signal at the 92nd percentile in both replicates (2902 genes)			
**REF6 target genes**	Gene dataset	At least one REF6-binding motif associated with a peak within the gene locus (3,054 genes)	3,072 genes	Cui et al., 2016 [3]	GSM5494309 GSM5494310
	ChIP-seq data	≥ 300bp with signal at the 92-95 percentile in both reps (777 genes)		He et al., 2021 [2]	
**H3K4me3**					GSM1225090
**H3K4me2**					DRX066756

References

1. Denoux, C.; Galletti, R.; Mammarella, N.; Gopalan, S.; Werck, D.; De Lorenzo, G.; Ferrari, S.; Ausubel, F.M.; Dewdney, J. Activation of Defense Response Pathways by OGs and Flg22 Elicitors in Arabidopsis Seedlings. Mol. Plant 2008, 1, 423–445.

2. He, K.; Mei, H.; Zhu, J.; Qiu, Q.; Cao, X.; Deng, X. The Histone H3K27 Demethylase REF6/JMJ12 Promotes Thermomorphogenesis in Arabidopsis. Natl. Sci. Rev. 2022, 9, nwab213.

3. Cui, X.; Lu, F.; Qiu, Q.; Zhou, B.; Gu, L.; Zhang, S.; Kang, Y.; Cui, X.; Ma, X.; Yao, Q.; et al. REF6 Recognizes a Specific DNA Sequence to Demethylate H3K27me3 and Regulate Organ Boundary Formation in Arabidopsis. Nat. Genet. 2016, 48, 694–699.
